# A multi-country comparison between mobile phone surveys and face-to-face household surveys to estimate the prevalence of non-communicable diseases behavioural risk factors in low- and middle-income settings

**DOI:** 10.1136/bmjgh-2024-017785

**Published:** 2025-06-25

**Authors:** Julián A Fernández-Niño, Saifuddin Ahmed, Gulam Muhammed Al Kibria, Stacy Davlin, Rachael Phadnis, Melanie Cowan, Romina Costa Beltrán, Juan Carlos Zevallos Lopez, Juan Vásconez, Jones K Masiye, Hicham El Berri, Samir Mounach, Mangla Gomare, Daksha Shah, Gulnar Khan, Niraj Dave, Champika Wickramasinghe, Udara Perera, Kennedy Lishimpi, Wilbroad Mutale, Namasiku Siyumbwa, Leanne Riley, Dustin G Gibson

**Affiliations:** 1 Department of International Health, Johns Hopkins Bloomberg School of Public Health, Baltimore, Maryland, USA; 2 Population, Family And Reproductive Health, Johns Hopkins University Bloomberg School of Public Health, Baltimore, Maryland, USA; 3 CDC Foundation Inc, Atlanta, Georgia, USA; 4 World Health Organization, Geneva, Switzerland; 5 Ministry of Public Health of Ecuador, Quito, Ecuador; 6 Government of Malawi Ministry of Health, Lilongwe, Malawi; 7 Government of the Kingdom of Morocco Ministry of Public Health, Rabat, Morocco; 8 Brihanmumbai Municipal Corporation, Mumbai, Maharashtra, India; 9 Nielsen India, Mumbai, Maharashtra, India; 10 Government of Sri Lanka Ministry of Health Sri Lanka, Colombo, Sri Lanka; 11 Zambia Ministry of Health, Lusaka, Zambia; 12 Center for Global Digital Health Innovation, Johns Hopkins Bloomberg School of Public Health, Baltimore, Maryland, USA

**Keywords:** Global Health, Cross-sectional survey, Epidemiology

## Abstract

**Background:**

Although mobile phone surveys (MPS) are routinely used to collect health information in high-income countries, concerns remain about the impact of bias on population-level estimates in low-income settings and validation studies are lacking. This study aims to compare non-communicable diseases (NCDs) risk factor estimates obtained from MPS and nationally representative face-to-face household surveys in six low- and middle-income settings.

**Methods:**

The MPS contained core questions from the standard STEPwise approach to NCD risk factor surveillance questionnaire. MPS sampling frames were generated by random digit dialling, while data collection was done by interactive voice response and SMS. At the same time, a nationally representative household survey (WHO STEPS) was conducted using multi-stage sampling. Participants aged 18 and older were included. Absolute differences and prevalence ratios, with 95% CIs, were analysed. The distribution of the differences between estimates by sex, age and education was also explored.

**Results:**

MPS and STEPS surveys were conducted in Ecuador, Malawi, Morocco, Zambia, Mumbai (India) and Sri Lanka between 2017 and 2022. Overall, MPS estimates of NCDs were most similar to STEPS estimates in Ecuador and Sri Lanka, and most dissimilar in Mumbai and Malawi. Broadly, smoking tobacco, fruit and vegetable consumption, and current drinking questions performed similarly across settings, whereas questions on smokeless tobacco, salt intake and hypertension yielded dissimilar results.

**Conclusions:**

MPS estimates were most similar to household estimates in settings with high levels of mobile phone ownership. MPS have the potential to serve as a valuable tool to monitor and address NCD risk factors, in addition to traditional face-to-face household surveys. However, producing nationally representative MPS estimates requires careful adjustments to sampling strategies, addressing coverage biases and overcoming technological limitations. Currently, face-to-face household surveys reach a more representative sample of the population, including those in remote and lower educational demographics.

**WHAT IS ALREADY KNOWN ON THIS TOPIC**

- Previous studies have shown that mobile phone surveys (MPS) have been effectively used in some low- and middle-income countries (LMICs) to collect information on behavioural risk factors of non-communicable diseases (NCDs) and during situations where face-to-face surveys were not feasible, though validation studies are lacking.
- While MPS and telephone surveys have been found to be an efficient option in high-income countries, concerns about potential biases affecting population-level estimates in LMICs have been raised.
- Coverage bias and non-response bias are significant challenges, potentially affecting the generalisability of findings and the applicability of MPS data for public health policy.
- Furthermore, biases in MPS may be exacerbated in LMICs due to factors such as institutional mistrust, lower familiarity with automated surveys and unequal distribution of mobile phone ownership among demographic subgroups.
- Previous research has indicated that certain groups, such as older individuals, rural populations, those with less education and disabled people, tend to be under-represented in MPS.
- However, there is a recognised need for comparative analyses at the national level between survey modalities in different countries and settings to explore the performance of MPS compared with traditional face-to-face surveys.

**WHAT THIS STUDY ADDS**

- This study is one of the first to compare health estimates collected from MPS and face-to-face interviews, specifically in the context of NCD risk factors across diverse LMICs.
- It contributes to the body of research by demonstrating how health indicators can vary considerably depending on the data collection method and socioeconomic context.
- Our research not only explores but also discusses potential explanations for these discrepancies, thus providing a more comprehensive understanding of how and why differences in estimates might occur.
- The insights garnered from this study are instrumental in guiding health surveillance methods and informing policy design and evaluation in LMIC settings for NCD.

**HOW THIS STUDY MIGHT AFFECT RESEARCH, PRACTICE OR POLICY**

- This research highlights the differences in health estimates obtained from MPS compared with face-to-face interviews, emphasising the importance of methodological considerations for NCD surveillance as crucial inputs for health policies and programmes.
- The study contributes to discussions on integrating technology in health surveillance, suggesting the potential for hybrid models that combine traditional and innovative methods.
- It also underscores the influence of socioeconomic factors on the surveillance of health data outcomes, emphasising the need for contextually relevant approaches.
- Furthermore, this study reinforces the necessity for international collaboration in developing effective health data collection strategies for NCDs in LMICs.

## Background

 Non-communicable diseases (NCDs) are a growing global health problem.^1^ In low- and middle-income countries (LMICs), their burden has significantly increased in the last decades, especially among the poorest.^2^ High-quality, reliable and nationally representative data are needed to inform NCD policies and public health interventions in these countries.^3 4^

National health surveys, when well designed and conducted, generate representative estimates on a population’s health status, which can be used to evaluate their needs for health services as well as the impact of public health policies.^5 6^ However, because of their significant costs, large logistical efforts and duration of field work demanded by face-to-face interviews,^7^ the availability of timely population data is limited, especially in resource-constrained settings.^5^ Given the increasing access to mobile phones in LMICs,^8^ mobile phone surveys (MPS) have emerged as a new method to collect health- and socioeconomic-related data in these countries,^9^ due to their lower cost and ability to quickly collect data.^7 10^

MPS have been previously used in some LMICs to collect information on behavioural risk factors for NCDs^11^ as well as more recently during COVID-19 and other humanitarian crises, where in-person household surveys were not feasible.^12 13^ Although MPS and telephone surveys have been found to be an efficient option to collect health information in high-income countries,^4 14 15^ there are some methodological concerns about the impact of bias on population-level estimates in LMICs and validation studies are lacking.^10^ Although coverage bias is also a challenge in face-to-face surveys, MPS may be more vulnerable to this bias at the sampling stage and non-response bias throughout data collection.^16 17^ Such biases could have significant implications for the generalisability of analyses undertaken and conclusions reached when using MPS survey data as an input for public health policies.^18 19^

Obtaining biased estimates using MPS may be even greater in LMICs.^20^ This is due to several factors, including uneven distribution of mobile phone ownership and access among demographic subgroups as well as higher institutional mistrust and lower familiarity with automated phone surveys. These factors may limit the representativeness of MPS in these countries.^10^ Previous studies have suggested that older, rural, less educated and disabled people tend to be under-represented in MPS.^21^ Considering the higher proportion of people in LMICs who live in rural areas and have a low educational level,^17^ this could affect the representativeness of estimations obtained in MPS in these countries.

To explore the performance of MPS in comparison to face-to-face household surveys, it is necessary to conduct comparative analyses at the national level between survey modalities in different countries and settings. In this study, we aimed to compare estimates of NCD behavioural risk factors between MPS and nationally representative household surveys in six LMICs: Ecuador, Malawi, Morocco, Mumbai (city of India), Sri Lanka and Zambia.

## Methods

### Data sources

For each country, we used information from two population-based surveys: the Mobile Phone Survey on NCDs (NCD MPS), supported by the US Centers for Disease Control and Prevention and the WHO STEPwise approach to NCD risk factor surveillance (STEPS), a face-to-face household survey. STEPS and NCD MPS surveys, respectively, were conducted in Ecuador (starting in May 2018 and January 2020), Malawi (October 2017 and April 2019), Morocco (March 2017 and January 2019), Mumbai, India (August and November 2021), Sri Lanka (April and December 2021) and Zambia (July and July 2017).

While the MPS were conducted at the national level in other study settings, in India, it was limited to Mumbai due to logistical and financial constraints. Mumbai was selected as it is India’s largest metropolitan area, with a highly heterogeneous population that shares many demographic and socioeconomic characteristics with the broader country. However, we acknowledge that this design limits the generalisability of the findings to the entire Indian population, particularly to rural areas where mobile phone ownership and usage patterns may differ. For simplicity, Mumbai will be referred to as a country, although both surveys were only representative of Mumbai city and not of India as a whole.

### Population of study and sampling

For NCD MPS, sampling frames for each country were generated using prefixes for each mobile network operator and random digit dialling (RDD) principles.^22^ Participants were enrolled if they were 18 years or older and the targeted quota for the sample size of the corresponding sex-age stratum had not been met, and consent to participate was given.^11^ In WHO STEPS, all countries drew nationally representative samples of adults aged 18–69 years (except Morocco, where the sample had no upper age limit) by means of a multi-stage sampling method. For comparison purposes, only data from step 1 and from participants aged 18–69 were included for analysis. Samples were weighted by age-sex distributions provided by each country. Country characteristics, including mobile phone ownership, literacy level, urbanicity and other demographics (online supplemental table 1) and sampling procedures (online supplemental tables 2 and 3) are provided.

### Procedures

Survey items used in the NCD MPS were extracted from STEPS instruments and adapted for mobile phone delivery. The survey included core questions (adapted by each country) on demographics, tobacco use, alcohol consumption, diet, diabetes and hypertension diagnoses and medication use. Data collection was done by means of interactive voice response (IVR) and SMS. Eligible participants were provided with a brief consent statement and were asked to press 1 if they agreed to participate in the study. MPS participants who completed the survey received airtime credit worth approximately US$1. Further details on the MPS have been described elsewhere.^11^

The WHO STEPS approach used a standardised method to collect, analyse and disseminate information on key NCD risk factors, enabling cross-country comparisons. Briefly, the WHO STEPS survey was conducted face-to-face by trained interviewers and had three steps: (1) collection of demographic information and NCD behavioural risk factors through self-report; (2) physical measurements; and (3) biochemical measurements. In this analysis, we only included NCD indicators from step 1 that were also assessed in the NCD MPS. Full methodology of WHO STEPS has been described elsewhere.^23 24^ Additional details on the NCD MPS and STEPS design and implementation are presented in online supplemental tables 2 and 3.

### Outcome variables

Unless otherwise specified, outcome variables were dichotomous (yes/no) and were either focused on NCD behavioural risk factors or NCD-related outcomes. Risk factor outcomes included (1) *tobacco use*: current and/or daily smoker and smokeless tobacco user; (2) *alcohol consumption*: current drinker (in the past 30 days) and heavy episodic drinking; (3) *diet*: mean number of days with fruit consumption a week, mean number of fruit servings consumed per day, mean number of days with vegetable consumption, mean number of vegetable servings consumed per day; less than five fruit or vegetable servings per day, adding salt during food preparation, adding salt while eating and eating processed food with high salt content. NCD-related outcomes included (1) hypertension: previous diagnosis and taking prescribed hypertension medication and (2) diabetes: previous diagnosis and taking prescribed diabetes medication.

### Statistical analysis

We first described the basic sociodemographic characteristics for the NCD MPS and STEPS in each country using age- and sex-weighted proportions and their respective 95% CI. Final disposition codes and outcome rates (Contact rate #1, Refusal rate #1, Cooperation rate #2 and Response rate #2) were calculated for NCD MPS using the standard definitions proposed by the American Association for Public Opinion Research.^25^ By survey mode, we calculated each indicator’s prevalence or mean with its corresponding 95% CI, considering the survey’s design effect.

We decided not to conduct statistical tests of the NCD indicator by survey mode because the large sample sizes allow for the significant detection of very small differences in survey estimates.^21^ Rather, the absolute difference (AD) of means and proportions for each NCD indicator was calculated and presented graphically. Considering that the ADs might not have the same meaning at different prevalence values, especially for very high or very low prevalence, we estimated the prevalence or means ratio and their respective 95% CI for all indicators. Finally, we compared the differences between countries in estimates obtained by NCD MPS versus STEPS when stratified by sex, age group and education level to explore the extent to which these differences, or similarities, between estimates remain among sub-populations. STEPS estimates were used as a reference for all comparisons.

### Role of the funding source

The funders of the study contributed to the overall study design, but had no role in data collection, data analysis, data interpretation or writing of the manuscript.

### Patient and public involvement

Patients or the public were involved in the design, or conduct, or reporting, or dissemination plans of our research. In 2015, experts in NCDs, mobile health and survey methodology selected questions from standardised surveys such as WHO STEPS and Tobacco Questions for Surveys to be included in the mobile phone survey. For each country, a series of key informant interviews, focus group discussions and user-group testing were conducted to identify appropriate examples to be used in the questions (ie, local examples of fruits and vegetables), to ensure the questionnaire was comprehensible and that the mobile phone survey platform was usable.

## Results

### Sample characteristics

Weighted sociodemographic characteristics of the participants in all six countries are shown in table 1. As per the study design, sex and age-group distributions were similar between surveys for each country. The proportion of females was slightly over 50% for both surveys in all countries with the exception of Mumbai (MPS: 45.7%; STEPS: 48.8%). Regarding age, Sri Lanka had the highest proportion of participants in the oldest age group, while Zambia and Malawi had higher proportions of the youngest age groups as compared with other countries.

**Table 1:** Sociodemographic characteristics of NCD MPS and WHO STEPS participants across settings

(A)	Ecuador		Sri Lanka		Morocco	
	MPS	STEPS	MPS	STEPS	MPS	STEPS
Sex						
Male	48.6 (46.8 to 50.4)	48.9 (47.2 to 50.6)	47.3 (45.8 to 48.8)	46.5 (45.0 to 48.0)	48.5 (46.5 to 50.5)	49.5 (47.9 to 51.1)
Female	51.4 (49.6 to 53.2)	51.1 (49.4 to 52.8)	52.7 (51.2 to 54.2)	53.5 (52.0 to 55.0)	51.5 (49.5 to 53.4)	50.5 (48.9 to 52.1)
Age (years)						
18–29	31.5 (29.9 to 33.2)	30.1 (28.4 to 31.8)	26.9 (26.8 to 27.1)	23.9 (22.5 to 25.4)	30.6 (28.9 to 32.3)	32.2 (30.6 to 33.9)
30–44	31.4 (29.8 to 33.0)	31.6 (29.9 to 33.2)	31.8 (31.6 to 32.0)	29.4 (28.1 to 30.7)	35.3 (33.3 to 37.1)	33.7 (32.2 to 35.2)
45–69	37.0 (35.3 to 38.8)	38.4 (36.5 to 40.3)	41.3 (41.0 to 41.7)	46.7 (45.2 to 48.2)	34.1 (32.2 to 36.0)	34.1 (32.7 to 35.5)
Education						
Up to primary	21.5 (19.4 to 23.6)	48.2 (44.9 to 51.7)	4.1 (3.2 to 4.9)	13.5 (12.1 to 15.1)	32.6 (28.3 to 36.8)	61.3 (58.6 to 64.1)
More than primary to up to secondary	43.7 (41.9 to 45.4)	36.2 (34.3 to 38.1)	36.4 (34.1 to 38.7)	62.0 (59.2 to 64.8)	41.1 (38.5 to 43.7)	27.7 (26.2 to 29.2)
More than secondary	34.8 (31.5 to 38.1)	15.6 (13.1 to 18.6)	59.5 (56.7 to 62.3)	24.5 (22.6 to 26.6)	26.3 (24.0 to 28.5)	11.0 (9.9 to 12.2)

(B)	Zambia		Mumbai		Malawi	
	MPS	STEPS	MPS	STEPS	MPS	STEPS
Sex						
Male	48.8 (47.3 to 50.3)	49.0 (47.3 to 50.7)	54.3 (52.7 to 55.9)	54.2 (52.6 to 55.8)	47.9 (46.4 to 49.4)	48.3 (44.3 to 52.3)
Female	51.2 (49.7 to 52.7)	51.0 (49.3 to 52.7)	45.7 (44.1 to 47.3)	45.8 (44.2 to 47.4)	52.1 (50.6 to 53.6)	51.7 (47.7 to 55.7)
Age (years)						
18–29	47.1 (45.6 to 48.5)	46.8 (44.8 to 48.9)	35.6 (33.5 to 37.7)	42.3 (40.3 to 44.4)	44.3 (42.8 to 45.7)	45.5 (42.9 to 48.1)
30–44	33.8 (32.5 to 35.2)	34.2 (32.3 to 36.2)	34.0 (32.5 to 35.5)	30.1 (28.6 to 31.6)	33.6 (32.2 to 35.0)	33.5 (31.2 to 35.8)
45–69	19.1 (17.8 to 20.5)	19.0 (17.6 to 20.4)	30.3 (28.3 to 32.3)	27.6 (25.7 to 29.7)	22.1 (20.4 to 23.8)	21.0 (18.8 to 23.4)
Education						
Up to primary	22.6 (20.0 to 25.2)	51.8 (48.0 to 56.0)	38.6 (35.9 to 41.3)	23.1 (19.9 to 26.7)	23.1 (21.2 to 25.1)	41.2 (36.8 to 46.0)
More than primary to up to secondary	36.8 (34.9 to 38.6)	39.9 (37.0 to 43.0)	16.2 (14.9 to 17.5)	31.4 (29.3 to 33.6)	56.4 (53.7 to 59.1)	55.7 (49.6 to 62.2)
More than secondary	40.6 (38.7 to 42.5)	8.3 (7.0 to 10.0)	45.3 (42.3 to 48.1)	45.5 (40.9 to 50.4)	20.5 (19.2 to 21.7)	3.0 (2.0 to 4.5)

Data are % (95% CI) and are weighted estimates for age-sex distribution of the population. Percentages do not all add up to 100% owing to rounding.

MPS, mobile phone survey; NCD MPS, Mobile Phone Survey on Non-Communicable Disease; STEPS, STEPwise approach to NCD risk factor surveillance.

By contrast, participants’ education level was different between the two surveys. In most countries, MPS respondents had higher education levels (more than primary education and more than secondary) compared with STEPS (38.6% vs 23.1%, respectively). Mumbai was the exception, where the proportion of participants with lower education (up to primary) in the MPS was higher than in the STEPS. Differences in education distribution were similar to the unweighted estimates (online supplemental table 4).

### Survey disposition codes and outcome rates

MPS response rates ranged from 3.1% (Mumbai) to 9.9% (Ecuador; table 2). STEPS response rates were higher than MPS and ranged from 69.4% (Ecuador) to 89.0% (Morocco; online supplemental table 3). MPS disposition codes were largely similar across countries with the exception of refusals (29.8%) and no answer (49.7%) for Sri Lanka, as compared with ranges of 0.08%–7.1% and 78.8%–80.3%, in other countries, respectively (online supplemental table 5).

**Table 2:** NCD MPS and WHO STEPS estimates of non-communicable diseases behavioural risk factors across countries and the city of Mumbai

(A)	Ecuador		Sri Lanka		Morocco	
Indicator	MPS	STEPS	MPS	STEPS	MPS	STEPS
Tobacco use						
Current smoker	15.5 (14.3 to 16.8)	13.7 (12.4 to 15.0)	15.3 (14.2 to 16.4)	14.1 (13.0 to 15.3)	20.4 (18.6 to 22.2)	12.3 (11.1 to 13.5)
Daily smoker	7.50 (6.62 to 8.49)	3.53 (2.89 to 4.32)	6.41 (3.46 to 4.68)	9.97 (9.87 to 11.9)	14.5 (12.9 to 16.1)	11.4 (10.3 to 12.6)
Current smokeless tobacco user	5.08 (4.34 to 5.95)	0.02 (0.00 to 0.11)	8.73 (7.85 to 9.61)	17.5 (16.2 to 18.8)	11.6 (10.0 to 13.2)	2.30 (1.80 to 2.90)
Daily smokeless tobacco users	1.96 (1.51 to 2.54)	0.00 (NE)	4.07 (8.51 to 10.3)	10.9 (17.3 to 19.9)	8.27 (6.90 to 9.60)	1.70 (1.20 to 2.20)
Alcohol consumption						
Current drinker (past 30 days)	36.7 (34.8 to 38.6)	39.3 (37.4 to 41.2)	22.0 (20.7 to 23.3)	20.7 (19.5 to 22.0)	8.04 (6.70 to 9.40)	1.90 (1.40 to 2.50)
Heavy episodic drinking	33.9 (32.0 to 35.7)	24.1 (22.5 to 25.8)	18.1 (16.9 to 19.3)	7.08 (6.23 to 7.92)	6.03 (4.80 to 7.30)	1.40 (1.00 to 1.90)
Diet (fruits and vegetables)						
Less than 5 fruit/veg. servings per day	91.3 (90.1 to 92.4)	94.6 (93.6 to 95.5)	78.7 (77.4 to 80.1)	67.9 (66.1 to 69.6)	72.9 (70.1 to 75.6)	76.3 (74.9 to 77.6)
Mean # of days fruits consumed per week	3.62 (3.54 to 3.70)	4.05 (3.95 to 4.15)	2.16 (2.08 to 2.24)	3.33 (3.20 to 3.46)	4.30 (4.20 to 4.40)	4.20 (4.20 to 4.30)
Mean # of fruit servings per day	1.16 (1.10 to 1.21)	1.11 (1.06 to 1.16)	1.52 (1.46 to 1.59)	1.24 (1.18 to 1.30)	1.83 (1.70 to 1.90)	1.00 (1.00 to 1.10)
Mean # of days veg. consumed per week	3.74 (3.66 to 3.81)	4.07 (3.98 to 4.16)	4.48 (4.42 to 4.54)	6.53 (6.49 to 6.57)	5.32 (5.20 to 5.40)	6.30 (6.30 to 6.40)
Mean # of veg. servings per day	1.18 (1.12 to 1.23)	0.88 (0.84 to 0.91)	3.30 (3.24 to 3.36)	3.45 (3.38 to 3.52)	2.53 (2.40 to 2.70)	2.50 (2.40 to 2.50)
Diet (salt intake)						
Add salt before/while eating	30.8 (29.0 to 32.7)	12.4 (11.1 to 13.9)	16.3 (15.2 to 17.5)	3.52 (2.85 to 4.18)	42.7 (39.4 to 45.9)	13.9 (12.8 to 15.0)
Add salt while preparing	41.8 (39.8 to 43.8)	76.3 (74.2 to 78.2)	62.2 (60.6 to 63.8)	NA	51.0 (47.6 to 54.4)	11.4 (10.4 to 12.4)
Eat processed foods high in salt	20.4 (18.8 to 22.0)	11.1 (9.99 to 12.4)	11.2 (10.2 to 12.2)	8.21 (7.25 to 9.17)	36.3 (33.1 to 39.4)	7.40 (6.50 to 8.30)
Medical conditions and treatment						
Diagnosis of hypertension	21.6 (19.9 to 23.3)	17.6 (16.3 to 19.1)	19.0 (17.7 to 20.2)	18.6 (17.5 to 19.7)	25.4 (22.3 to 28.4)	11.0 (10.1 to 11.8)
Treatment for hypertension	46.8 (42.4 to 51.3)	44.9 (40.8 to 49.1)	61.8 (58.1 to 65.4)	59.4 (56.2 to 62.7)	34.2 (27.4 to 41.0)	40.1 (36.3 to 43.9)
Diagnosis of diabetes	12.2 (10.9 to 13.6)	6.57 (5.69 to 7.58)	16.8 (15.6 to 17.9)	13.9 (12.9 to 14.8)	8.07 (6.00 to 10.1)	6.30 (5.70 to 7.00)
Treatment for diabetes	41.8 (36.0 to 47.9)	51.1 (44.5 to 57.7)	66.5 (62.8 to 70.3)	72.2 (68.8 to 75.6)	53.0 (39.4 to 66.7)	70.2 (65.1 to 74.8)

(B)	Zambia		Mumbai		Malawi	
Indicator	MPS	STEPS	MPS	STEPS	MPS	STEPS
Tobacco use						
Current smoker	16.4 (14.9 to 18.0)	12.3 (11.0 to 13.7)	24.9 (23.4 to 26.3)	6.30 (5.05 to 7.55)	22.8 (21.5 to 24.1)	11.3 (8.98;14.0)
Daily smoker	5.44 (4.60 to 6.30)	9.00 (8.00 to 10.2)	11.2 (9.54 to 11.7)	4.31 (7.24 to 9.57)	4.42 (3.89 to 5.03)	8.38 (6.40 to 10.9)
Current smokeless tobacco user	16.3 (14.8 to 17.8)	4.50 (3.80 to 5.40)	20.0 (18.6 to 21.4)	10.7 (9.49 to 11.9)	18.1 (16.9 to 19.4)	1.39 (1.00 to 1.94)
Daily smokeless tobacco users	5.22 (4.30 to 6.10)	2.10 (2.60 to 2.60)	10.6 (14.0 to 16.5)	8.4 (10.5 to 13.2)	3.98 (3.42 to 4.62)	0.75 (0.53 to 1.06)
Alcohol consumption						
Current drinker (past 30 days)	31.1 (29.3 to 33.0)	21.7 (20.1 to 23.5)	31.2 (29.3 to 33.0)	6.59 (5.63 to 7.56)	16.9 (15.8 to 18.1)	17.3 (14.5 to 20.5)
Heavy episodic drinking	27.4 (25.6 to 29.2)	10.9 (9.60 to 12.2)	27.0 (25.2 to 28.8)	3.06 (2.42 to 3.70)	14.7 (13.6 to 15.8)	7.80 (6.64 to 9.15)
Diet (fruits and vegetables)						
Less than 5 fruit/veg. servings per day	76.1 (74.3 to 78.0)	90.4 (88.4 to 92.0)	79.0 (77.5 to 80.6)	94.1 (92.5 to 95.8)	54.2 (52.4 to 55.9)	90.3 (87.9 to 92.2)
Mean # of days fruits consumed per week	2.83 (2.80 to 2.90)	2.10 (1.90 to 2.20)	3.16 (3.09 to 3.23)	4.06 (3.95 to 4.18)	3.64 (3.57 to 3.71)	2.47 (2.22 to 2.71)
Mean # of fruit servings per day	1.33 (1.20 to 1.40)	0.70 (0.60 to 0.80)	1.36 (1.29 to 1.42)	0.91 (0.87 to 0.95)	1.73 (1.67 to 1.79)	0.87 (0.74 to 1.00)
Mean # of days veg. consumed per week	5.53 (5.40 to 5.60)	6.30 (6.20 to 6.30)	4.35 (4.27 to 4.42)	5.65 (5.56 to 5.73)	5.13 (5.07 to 5.20)	5.18 (5.00 to 5.35)
Mean # of veg. servings per day	2.80 (2.60 to 2.90)	2.14 (2.00 to 2.30)	2.33 (2.23 to 2.42)	1.70 (1.59 to 1.80)	3.73 (3.64 to 3.82)	1.80 (1.68 to 1.92)
Diet (salt intake)						
Add salt before/while eating	56.3 (54.1 to 58.5)	39.8 (37.0 to 42.5)	24.3 (22.8 to 25.9)	7.24 (5.96 to 8.51)	40.9 (39.2 to 42.5)	17.4 (14.4 to 20.9)
Add salt while preparing	82.4 (80.7 to 84.1)	81.5 (79.3 to 83.7)	27.4 (25.8 to 29.1)	15.7 (12.7 to 18.6)	59.2 (57.5 to 61.0)	63.7 (59.4 to 67.8)
Eat processed foods high in salt	34.9 (32.7 to 37.0)	6.00 (5.00 to 7.10)	15.0 (13.8 to 16.3)	10.2 (8.48 to 11.9)	32.5 (30.9 to 34.1)	38.2 (33.4 to 43.1)
Medical conditions and treatment						
Diagnosis of hypertension	23.8 (21.8 to 25.7)	11.7 (10.4 to 13.1)	13.3 (12.0 to 14.5)	5.51 (4.66 to 6.36)	23.8 (22.2 to 25.4)	7.98 (6.74 to 9.42)
Treatment for hypertension	35.2 (30.3 to 40.2)	26.0 (21.5 to 31.2)	56.8 (51.6 to 61.9)	64.2 (57.5 to 70.9)	32.7 (28.7 to 37.0)	30.3 (23.7 to 37.7)
Diagnosis of diabetes	6.19 (5.00 to 7.40)	1.60 (1.20 to 2.20)	9.02 (7.94 to 10.1)	3.36 (2.69 to 4.03)	4.68 (3.92 to 5.58)	0.70 (0.39 to 1.26)
Treatment for diabetes	44.8 (34.4 to 55.3)	36.8 (23.7 to 52.1)	71.0 (65.1 to 76.9)	78.7 (70.6 to 86.7)	33.4 (24.6 to 43.5)	47.9 (22.6 to 74.3)

Data are % (95% CI) and weighted estimates.

NA, non-available data to NCD MPS, Mobile Phone Survey on Non-Communicable Disease; NE, Non-estimable; STEPS, STEPwise approach to NCD risk factor surveillance.

### Comparison of NCD MPS and WHO STEPS prevalence estimates: NCD and behavioural risk factors

**Figure 1:**
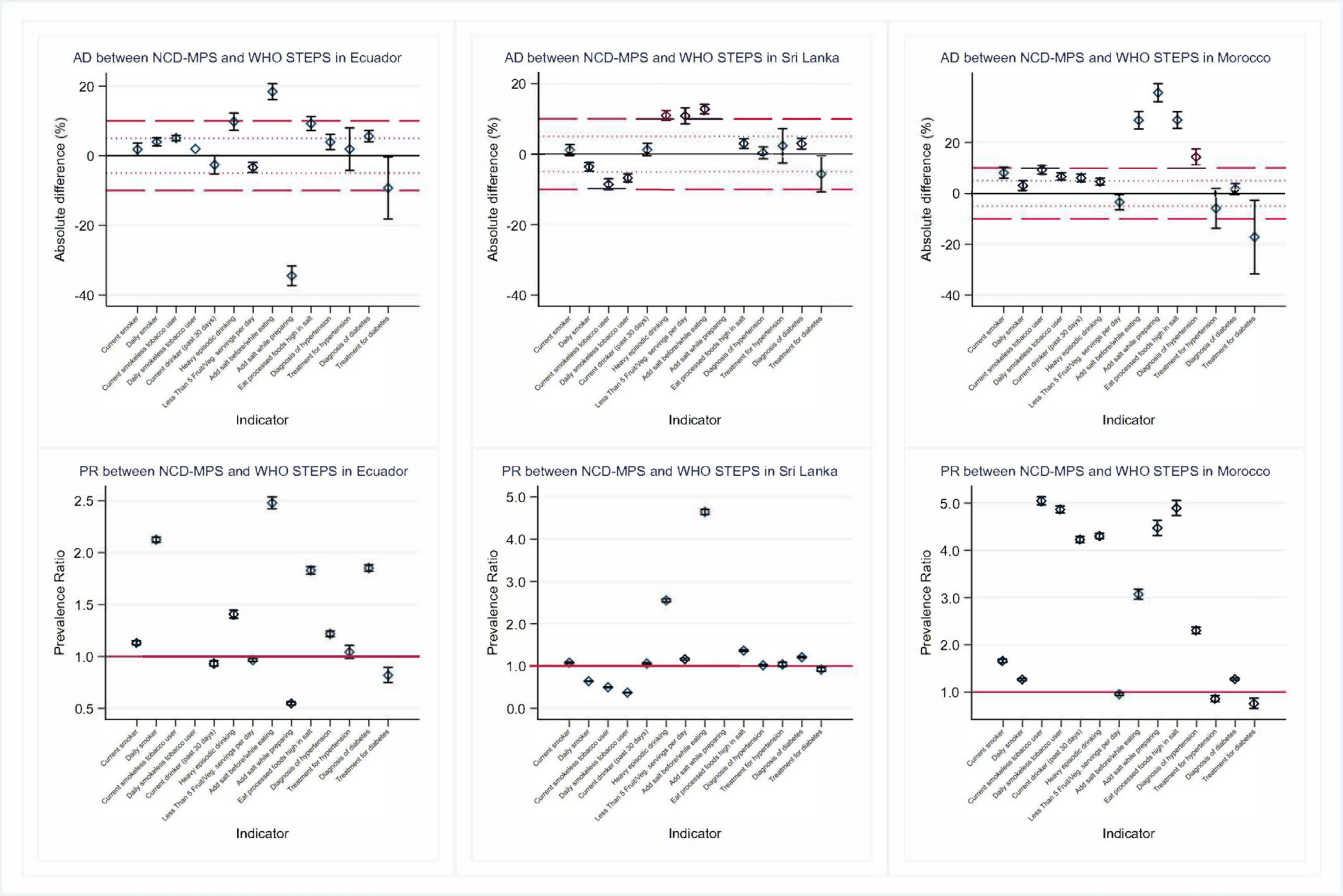
Absolute difference and prevalence ratio between NCD MPS and WHO STEPS for all non-communicable diseases behavioural risk factors for Ecuador, Sri Lanka and Morocco. Prevalence ratio not shown for current smokeless tobacco use in Ecuador because it dramatically affects the plot. NCD MPS, Mobile Phone Survey on Non-Communicable Disease; STEPS, STEPwise approach to NCD risk factor surveillance.

Broadly, some key tobacco indicators (ie, current smokers, daily smokers and daily smokeless tobacco use indicators) had higher prevalence estimates in the NCD MPS than STEPS, except for Sri Lanka and for the daily smoker indicator in Zambia and Malawi (table 3). For current smokers, survey estimates were similar in Ecuador, Sri Lanka and Zambia; with MPS reporting higher estimates than STEPS in Morocco (AD=8.10%, 95% CI 5.92% to 10.3%), Mumbai (AD=18.6%; 95% CI 16.7% to 20.5%) and Malawi (AD=11.5%; 95% CI 8.7% to 14.3%; online supplemental table 4). Although daily smoking estimates were within ±5% points for all countries except Mumbai, PRs indicate a near doubling in estimates between surveys (figures1  2). The current smokeless tobacco indicator had ADs greater than 5% for all countries, with large prevalence ratios (PRs), particularly for Ecuador (PR=254; 95% CI 252 to 256), Morocco (PR=5.04; 95% CI 4.96 to 5.13), Zambia (PR=3.63; 95% CI 3.57 to 3.69) and Malawi (PR=13.0; 95% CI 12.7 to 13.2). Daily smokeless tobacco use performed like current smokeless tobacco use.

**Figure 2:**
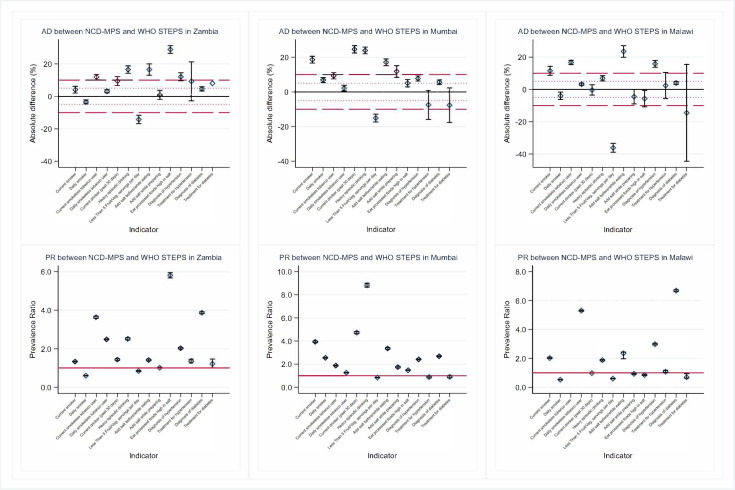
Absolute difference and prevalence ratio between NCD MPS and WHO STEPS for all non-communicable diseases behavioural risk factors for Zambia, Mumbai and Malawi. Prevalence ratio not shown for current smokeless tobacco use in Malawi because it would dramatically affect the plot. The 95% CI for the absolute difference of treatment for diabetes in Zambia was also dropped for the same reason. NCD MPS, Mobile Phone Survey on Non-Communicable Disease; STEPS, STEPwise approach to NCD risk factor surveillance.

**Table 3:** Absolute difference and ratio of estimates between NCD MPS and WHO STEPS estimates across countries and the city of Mumbai

(A)	Ecuador		Sri Lanka		Morocco	
Indicator	Absolute difference	Prevalence ratio*	Absolute difference	Prevalence ratio*	Absolute difference	Prevalence ratio*
Tobacco use						
Current smokers	1.80 (−0.01 to 3.60)	1.13 (1.11 to 1.15)	1.15 (−0.41 to 2.71)	1.08 (1.06 to 1.10)	8.10 (5.92 to 10.3)	1.66 (1.62 to 1.69)
Daily smokers	3.97 (2.80 to 5.14)	2.12 (2.10 to 2.15)	−3.56 (−7.99 to −5.63)	0.64 (0.37 to 0.38)	3.10 (1.14 to 5.05)	1.27 (1.25 to 1.30)
Current smokeless tobacco users	5.06 (4.25 to 5.86)	254 (252 to 256)	−8.76 (−10.3 to −7.22)	0.50 (0.49 to 0.51)	9.30 (7.58 to 11.0)	5.04 (4.96 to 5.13)
Daily smokeless tobacco use	1.96 (NE)	NE (NE)	−6.81 (−10.8 to −7.57)	0.37 (0.50 to 0.51)	6.57 (5.11 to 8.02)	4.86 (4.79 to 4.93)
Alcohol consumption						
Current drinker (past 30 days)	−2.62 (−5.28 to 0.05)	0.93 (0.91 to 0.96)	1.24 (−0.56 to 3.04)	1.06 (1.04 to 1.08)	6.14 (4.66 to 7.61)	4.23 (4.17 to 4.29)
Heavy episodic drinking	9.76 (7.30 to 12.2)	1.41 (1.37 to 1.44)	11.0 (9.56 to 12.5)	2.55 (2.52 to 2.59)	4.63 (3.30 to 5.97)	4.31 (4.25 to 4.37)
Diet (fruit and vegetables)						
Less than 5 fruit/veg. servings per day	−3.33 (−4.78 to −1.87)	0.96 (0.95 to 0.98)	10.9 (8.64 to 13.1)	1.16 (1.13 to 1.19)	−3.43 (−6.48 to −0.38)	0.96 (0.93 to 0.98)
Mean # days fruit consumed per week*	−0.43 (−0.55 to −0.30)	0.89 (0.79 to 1.01)	−1.17 (−1.32 to −1.02)	0.65 (0.56 to 0.75)	0.11 (−6.97 to 7.19)	1.03 (0.96 to 1.10)
Mean # fruit servings per day*	0.05 (−0.02 to −0.12)	1.04 (0.97 to 1.12)	0.29 (0.20 to 0.37)	1.23 (1.13 to 1.34)	0.83 (−4.07 to 5.73)	1.83 (1.74 to 1.92)
Mean # days veg. consumed per week*	−0.33 (−0.45 to −0.21)	0.92 (0.82 to 1.03)	−2.05 (−2.12 to −1.97)	0.69 (0.64 to 0.74)	−0.98 (−5.08 to 3.12)	0.84 (0.81 to 0.88)
Mean # veg. servings per day*	0.30 (0.24 to 0.36)	1.34 (1.26 to 1.43)	−0.15 (−0.24 to −0.05)	0.96 (0.87 to 1.05)	0.03 (−4.91 to 4.97)	1.01 (0.96 to 1.06)
Diet (salt intake)						
Add salt before/while eating	18.38 (16.1 to 20.7)	2.48 (2.42 to 2.54)	12.8 (11.5 to 14.2)	4.65 (4.58 to 4.71)	28.8 (25.3 to 32.2)	3.07 (2.97 to 3.18)
Add salt while preparing	−34.5 (−37.3 to −31.6)	0.55 (0.53 to 0.56)	NE (NE)	NE (NE)	39.6 (36.0 to 43.1)	4.47 (4.31 to 4.63)
Eat processed foods high in salt	9.22 (7.22 to 11.2)	1.83 (1.79 to 1.87)	3.01 (1.62 to 4.40)	1.37 (1.35 to 1.39)	28.9 (25.6 to 32.1)	4.90 (4.74 to 5.06)
Medical conditions and treatment						
Diagnosis of hypertension	3.94 (1.74 to 6.13)	1.22 (1.19 to 1.24)	0.36 (−1.33 to 2.05)	1.02 (1.00 to 1.04)	14.4 (11.2 to 17.5)	2.30 (2.23 to 2.38)
Treatment for hypertension	1.90 (−4.19 to 7.99)	1.04 (0.98 to 1.11)	2.36 (−2.53 to 7.25)	1.04 (0.99 to 1.09)	−5.90 (−13.7 to 1.92)	0.85 (0.79 to 0.92)
Diagnosis of diabetes	5.59 (3.95 to 7.23)	1.85 (1.82 to 1.88)	2.90 (1.37 to 4.43)	1.21 (1.19 to 1.23)	1.77 (−0.37 to 3.91)	1.28 (1.25 to 1.31)
Treatment for diabetes	−9.27 (−18.2 to −0.37)	0.82 (0.75 to 0.89)	−5.66 (−10.7 to −0.62)	0.92 (0.88 to 0.97)	−17.2 (−31.7 to −2.70)	0.76 (0.65 to 0.87)

(B)	Zambia		Mumbai		Malawi	
Indicator	Absolute difference	Prevalence ratio*	Absolute difference	Prevalence ratio*	Absolute difference	Prevalence ratio*
Tobacco use						
Current smokers	4.14 (2.08 to 6.21)	1.34 (1.31 to 1.37)	18.6 (16.7 to 20.5)	3.95 (3.88 to 4.03)	11.5 (8.7 to 14.3)	2.02 (2.00 to 2.08)
Daily smokers	−3.56 (−4.80 to −2.33)	0.60 (0.60 to 0.61)	6.84 (0.62 to 3.79)	2.56 (1.24 to 1.28)	−3.96 (−6.25 to −1.66)	0.53 (0.51 to 0.54)
Current smokeless tobacco users	11.8 (10.2 to 13.6)	3.63 (3.57 to 3.69)	9.29 (7.45 to 11.1)	1.87 (1.83 to 1.90)	16.7 (15.4 to 18.0)	13.0 (12.7 to 13.2)
Daily smokeless tobacco use	3.12 (2.18 to 4.07)	2.49 (2.46 to 2.51)	2.21 (1.54 to 5.36)	1.27 (1.27 to 1.32)	3.22 (2.58 to 3.87)	5.28 (5.27 to 5.34)
Alcohol consumption						
Current drinker (past 30 days)	9.41 (6.67 to 12.1)	1.43 (1.40 to 1.47)	24.6 (22.5 to 26.7)	4.73 (4.63 to 4.83)	−0.32 (−3.51 to 2.87)	0.98 (0.97 to 1.01)
Heavy episodic drinking	16.5 (14.2 to 18.7)	2.51 (2.46 to 2.57)	23.9 (22.0 to 25.9)	8.83 (8.66 to 9.00)	6.9 (5.23 to 8.50)	1.88 (1.82 to 1.91)
Diet (fruit and vegetables)						
Less than 5 fruit/veg. servings per day	−14.3 (−16.8 to −11.7)	0.84 (0.82 to 0.86)	−15.1 (−17.4 to −12.8)	0.84 (0.82 to 0.86)	−36.1 (−38.9 to −33.4)	0.60 (0.59 to 0.62)
Mean # days fruit consumed per week*	0.73 (−1.08 to 2.53)	1.35 (1.24 to 1.46)	−0.91 (−1.04 to −0.77)	0.78 (0.68 to 0.89)	1.17 (0.92 to 1.42)	1.47 (1.28 to 1.89)
Mean # fruit servings per day*	0.63 (−1.17 to 2.44)	1.91 (1.74 to 2.09)	0.45 (0.37 to 0.52)	1.49 (1.38 to 1.61)	0.86 (0.72 to 1.00)	1.99 (1.64 to 2.29)
Mean # days veg. consumed per week*	−0.77 (−2.58 to 1.04)	0.88 (0.81 to 0.95)	−1.30 (−1.41 to −1.18)	0.77 (0.69 to 0.86)	−0.05 (−0.23 to 0.15)	0.99 (0.77 to 1.19)
Mean # veg. servings per day*	0.66 (−1.15 to 2.47)	1.32 (1.17 to 1.48)	0.63 (0.49 to 0.77)	1.37 (1.19 to 1.58)	1.92 (1.77 to 2.08)	2.07 (1.77 to 2.41)
Diet (salt intake)						
Add salt before/while eating	16.5 (13.0 to 20.0)	1.41 (1.37 to 1.47)	17.1 (15.1 to 19.1)	3.36 (3.30 to 3.43)	23.5 (19.9 to 27.1)	2.35 (1.97 to 2.43)
Add salt while preparing	0.89 (−1.88 to 3.66)	1.01 (0.98 to 1.04)	11.8 (8.41 to 15.1)	1.75 (1.69 to 1.81)	−4.50 (−9.00 to 0.00)	0.93 (0.90 to 0.97)
Eat processed foods high in salt	28.9 (26.5 to 31.2)	5.81 (5.67 to 5.95)	4.87 (2.75 to 7.00)	1.48 (1.45 to 1.51)	−5.7 (−10.8 to −0.6)	0.85 (0.81 to 0.90)
Medical conditions and treatment						
Diagnosis of hypertension	12.1 (9.59 to 14.5)	2.03 (1.98 to 2.08)	7.75 (6.23 to 9.27)	2.41 (2.37 to 2.44)	15.8 (13.7to 17.9)	2.98 (2.93 to 3.04)
Treatment for hypertension	9.24 (−2.75 to 21.2)	1.36 (1.26 to 1.46)	−7.45 (−15.9 to 0.96)	0.88 (0.81 to 0.96)	2.40 (−5.7 to 10.5)	1.08 (1.03 to 1.17)
Diagnosis of diabetes	4.59 (3.29 to 5.89)	3.87 (3.82 to 3.92)	5.66 (4.39 to 6.92)	2.68 (2.65 to 2.72)	4.00 (3.10 to 4.90)	6.69 (6.63 to 6.75)
Treatment for diabetes	8.01 (−63.4 to 79.4)	1.22 (1.01 to 1.47)	−7.66 (−17.6 to 2.31)	0.90 (0.82 to 1.00)	−14.5 (−44.5 to 15.5)	0.70 (0.63 to 0.94)

Data are differences (95% CI) or ratios (95%). WHO STEPS is a reference survey.

* Means ratio NE.

NCD MPS, Mobile Phone Survey on Non-Communicable Disease; NE, non-estimable; STEPS, STEPwise approach to NCD risk factor surveillance.

The proportion of current drinkers estimated by both surveys was similar in Ecuador, Sri Lanka and Malawi. NCD MPS estimates were higher than STEPS in Mumbai (31.2% vs 6.59%, PR=4.73; 95% CI 4.63 to 4.83) and Morocco (8.4% vs 1.9%, PR=4.23; 95% CI 4.17 to 4.29). For the proportion of heavy episodic drinkers, estimates derived from NCD MPS were higher than STEPS for all countries. The differences between survey estimates for heavy episodic drinking had PRs that ranged from 1.41 (MPS p=33.9%, STEPS p=24.1%) in Ecuador to 8.83 (MPS p=27.0%, STEPS p=3.06%) in Mumbai.

The mean days eating fruits and vegetables survey estimates were within 1 day absolute difference for all countries except Sri Lanka (fruit days AD=−1.17, vegetable days AD=−2.05), Malawi (fruit days AD=1.17) and Mumbai (vegetable days AD=−1.30). Similarly, mean servings of fruits and mean servings of vegetables were within an absolute difference of 1.0 for all countries except for Malawi (vegetable servings AD=1.92). Regarding salt consumption, MPS estimates were higher than STEPS for all three indicators across all six countries except in Ecuador (adding salt while preparing food AD=−34.5%) and Malawi (adding salt while preparing food AD=−4.50% and eating processed foods AD=−5.7%). Absolute differences for adding salt while eating and adding salt while preparing food ranged from 12.8% in Sri Lanka to 28.8% in Morocco and from −34.5% in Ecuador to 39.6% in Morocco, respectively.

Finally, the estimated prevalence of self-reported hypertension and self-reported diabetes diagnoses was always higher in NCD MPS than in STEPS. Both indicators had PRs greater than 2.0 in Morocco (hypertension only), Zambia, Mumbai and Malawi. Hypertension and diabetes diagnoses were similar between surveys in Ecuador and Sri Lanka. Hypertension diagnoses had PRs less than 1.3 and ADs less than 5% in Ecuador and Sri Lanka. Diabetes diagnosis PRs were less than 1.3 in Sri Lanka (PR=1.21; 95% CI 1.19 to 1.23) and Morocco (PR=1.28; 95% CI 1.25 to 1.31).

### Exploration by sex, age and education

Although no systematic differences in survey estimates were observed when stratifying results by sex or age (online supplemental figures 1–12), the extent of these differences varied across countries. For most indicators, there were no apparent trends across education levels except for those related to the use of salt, particularly adding salt while cooking and adding salt while eating, where the AD tended to be lower as the level of education increased (online supplemental figures 13–18). Both the prevalence of hypertension and diabetes had similar patterns.

## Discussion

This is one of the first studies to compare health estimates collected from MPS and face-to-face interviews, using nationally representative surveys conducted across diverse LMIC settings. Our analysis focused on key indicators of NCD risk factors which provide valuable insights for informing health policy design and evaluation. Overall, NCD MPS estimates were most similar to STEPS estimates in Ecuador and Sri Lanka, and most dissimilar in Mumbai and Malawi. Although the small number of countries in our study does not allow for making global inferences, the two countries with the highest socioeconomic and macro health indicators were also the same countries which showed the least differences in survey estimates. The association of income level with indicator agreement between survey modalities has been previously reported.^21^

Similar studies have also compared the performance of MPS versus face-to-face surveys in other LMICs such as Burkina Faso,^26^ Sub-Saharan Africa,^27^ Lebanon^28^ and Ghana.^18^ In Ghana, a similar methodological approach was used to compare media use and bed-net use estimates derived from an RDD IVR survey and the Demographic and Health Survey.^18^ In contrast to our results, in Lebanon, no differences were observed for all NCD indicators when comparing estimates from cell phone owners and owners of any telephone (landline and/or cell phone) to the overall household sample, except for binge drinking.^28^ However, the differences found between the countries included in our study make evident the difficulties in comparing our findings with those of other comparative reports from other countries due to differences in the socioeconomic context that could affect the MPS performance.

This study also uncovered significant absolute and relative discrepancies in estimates in many indicators between survey modalities, with most estimates notably higher in NCD MPS. Broadly speaking, smoking tobacco, fruit and vegetable consumption, and current drinking questions performed similarly across settings, whereas questions on smokeless tobacco, salt intake and hypertension yielded dissimilar results. Moreover, the variance of the NCD indicator estimates was low due to a large sample size. Consequently, beyond the analysis of the magnitude of the absolute or relative differences, it is also necessary to determine if the differences in the estimates between survey modalities would produce real changes in the policies in each context and problem (ie, if over 90% of the population does not eat enough fruits and vegetables, does an AD of only 3.3% between estimates change how this issue is addressed?). However, for monitoring changes in indicators over time, both the accuracy and precision of the estimators are highly relevant. Thus, the impact of bias could indeed have significance from a public health perspective.

Differences between NCD MPS and STEPS estimates can arise from various reasons: (1) variations in question formats, such as the absence of a tobacco screening question in NCD MPS; (2) a low prevalence of some indicators affecting statistical power to compare estimates; (3) structural country features like mobile phone usage, mobile phone infrastructure, urbanisation, adult literacy and GDP per capita; (4) different national representativeness: MPS lacks the ability to geographically pinpoint participants, unlike face-to-face surveys; (5) the desire for social approval may vary based on survey type,^29^ especially regarding sensitive topics like alcohol consumption and tobacco use; and (6) exogenous variables, such as socioeconomic status, leading to selection bias: discrepancies between NCD MPS and STEPS in this study likely stem from the MPS sample having sociodemographic traits, other than age and sex which were controlled for, distinct from the STEPS sample and are related to specific NCD indicators.^30 31^ For instance, MPS participants possess higher educational levels, which often correlates with socioeconomic status and mobile phone ownership.^2132^,^35^ This education can influence one’s likelihood to respond to MPS, which might also correlate with certain health outcomes studied. Despite adjustments in the data, significant educational distribution differences persisted across surveys. Coverage bias in MPS is more magnified in countries with lower levels of mobile phone ownership. It is likely that non-response bias is also higher in MPS than in STEPS, but its magnitude is unknown.

The use of post-stratification weights to estimate the indicators is addressed to make the estimates more demographically representative of the general population.^18 36^ Although this approach may improve the estimates made by improving the demographic representation of the sample, it implicitly assumes there are no differences between those with and without access to mobile phones, and therefore does not address coverage bias.^16^ As the differences in the distribution of education show, there are differences in the population selected (beyond sex and age) that partially explain the observed differences in the estimates between surveys. Similarly, previous studies have documented that both SMS and IVR tend to produce biased estimates compared with official statistics in other fields (eg, voting surveys), and have also demonstrated that weighting for demographic characteristics does not always reduce this bias.^37^ However, the small sample size within each substratum in this study makes it difficult to assess the influence of education level and other socioeconomic factors on selection bias due to non-coverage and non-response at the individual level. It is also possible that for people with a higher educational level, the estimates between both survey modalities tend to converge more where there is greater response, acceptance and understanding of the questions. This was evident in our exploratory analysis in certain indicators, such as salt consumption, where the ADs tended to be reduced with a higher educational level. More efforts are needed to make MPS more representative at a national level in LMICs where these surveys would be more useful in real-world conditions.

Our study has several limitations. First, surveys were conducted in only six countries and our findings may not be generalisable to other countries. Additionally, the MPS in India was restricted to Mumbai. While Mumbai is India’s largest metropolitan area and provides valuable insights due to its demographic diversity, findings may not be fully representative of the broader Indian population, especially in rural areas with different mobile phone usage patterns and accessibility. Future studies should explore the feasibility of expanding MPS across different regions of India to improve national representativeness. Second, data collection for NCD MPS and STEPS was not simultaneous, occurring between 2017 and 2022. This timeframe means the surveys took place at varying points relative to the COVID-19 pandemic across different countries and survey types (ranging from both surveys pre-pandemic in Zambia to both surveys during the pandemic in Sri Lanka and Mumbai). This lack of consistent timing relative to the pandemic limits direct comparability. Additionally, pandemic-related behavioural changes might have influenced NCD risk factor prevalence in surveys conducted from 2020 onwards. Evidence on the impact of the pandemic on alcohol and tobacco use is mixed, with some studies reporting increased consumption, particularly in high-income settings, while others suggest stable or decreased use due to health concerns or restricted availability during lockdowns.^38 39^ While we account for these differences, future studies should consider further refining methodological strategies to account for pandemic-related behavioural variations.

Third, there were small sample sizes across specific age-sex-education level strata. Consequently, despite attempting to control the selection bias by means of stratification, only partial adjusting was achieved due to (1) the presence of variables related to participation in the surveys that were not measured/identified and thus could not be controlled for and (2) the sample size not being able to provide enough statistical power to identify bias.

Additionally, MPS may be more vulnerable to coverage and non-response biases, as mobile phone access varies by age, gender, socioeconomic status and geographic location. These biases could have led to the under-representation of specific populations, particularly those with lower mobile phone penetration, affecting the generalisability of findings. Furthermore, we acknowledge the potential for social desirability bias, particularly in self-reported behaviours such as alcohol consumption and smoking. The difference in data collection methods—IVR/SMS (MPS) versus face-to-face interviews (STEPS)—may have influenced response patterns, leading to potential underreporting in some settings. Future research should explore strategies to mitigate these biases, such as mixed-method approaches that integrate qualitative assessments to better understand respondent behaviour in different survey modes.

Despite these limitations, our findings offer valuable insights into the nature of survey discrepancies. The discrepancies in these estimates are likely due to the different impacts of selection bias, socioeconomic differences in the populations reached by each modality and the limitations of our study. This exploratory research is designed primarily to highlight these differences, encouraging more detailed investigations using specific methodologies to delve into the sources of selection bias.

## Conclusions

The findings have implications for conducting public health surveillance to provide data for supporting public health policies and interventions targeting NCDs in LMICs. The results demonstrate that while MPS have the potential to serve as a valuable tool alongside traditional face-to-face household surveys to monitor and address NCD risk factors, household surveys currently reach a more representative sample of the population, including those remote and underserved populations. In settings with high mobile phone penetration, MPS can provide timely and cost-effective data on a limited set of NCD indicators, complementing the more resource-intensive face-to-face household surveys, which have the advantage of collecting a more comprehensive and detailed set of indicators and physical measurements. To effectively use the results from MPS alongside household surveys, further research into their application at the country level is necessary. This may involve conducting further validation studies and adjusting sampling strategies of the MPS to ensure better representativeness across different demographic subgroups.

## Supplementary material

10.1136/bmjgh-2024-017785 online supplemental file 1

## Data Availability

Data are available upon reasonable request.
