# Supplementary material for: A multi-country comparison between mobile phone surveys and face-to-face household surveys to estimate the prevalence of non-communicable diseases behavioural risk factors in low- and middle-income settings
Source: BMJ Glob Health. 2025 Jun 25;10(6):e017785. doi: 10.1136/bmjgh-2024-017785 (PMC12198783; doi:10.1136/bmjgh-2024-017785)

**Supplementary Materials**

Contents

[Supplementary Table 1. Comparison of Socioeconomic Characteristics across four studied countries 2](#_Toc199146812)

[**Supplementary Table 2. Comparison of NCD-MPS design and implementation across studied countries** 3](#_Toc199146813)

[**Supplementary Table 3. Comparison of WHO STEPS design and implementation across studied countries** 4](#_Toc199146814)

[**Supplementary Table 4. Sociodemographic characteristics of NCD-MPS and WHO STEPS participants across countries and the city of Mumbai (unweighted estimates)** 5](#_Toc199146815)

[**Supplementary Table 5. Final disposition case codes and outcome rates for dialed phone numbers in mobile phone survey across countries and the city of Mumbai** 6](#_Toc199146816)

[**Supplementary Figure 1. Subgroup analysis of NCD indicators by sex in Ecuador** 7](#_Toc199146817)

[**Supplementary Figure 2. Subgroup analysis of NCD indicators by sex in Malawi** 8](#_Toc199146818)

[**Supplementary Figure 3. Subgroup analysis of NCD indicators by sex in Morocco** 9](#_Toc199146819)

[**Supplementary Figure 4. Subgroup analysis of NCD indicators by sex in Zambia** 10](#_Toc199146820)

[**Supplementary Figure 5. Subgroup analysis of NCD indicators by sex in Sri Lanka** 11](#_Toc199146821)

[**Supplementary Figure 6. Subgroup analysis of NCD indicators by sex in Mumbai** 12](#_Toc199146822)

[**Supplementary Figure 7. Subgroup analysis of NCD indicators by age in Ecuador** 13](#_Toc199146823)

[**Supplementary Figure 8. Subgroup analysis of NCD indicators by age in Malawi** 14](#_Toc199146824)

[**Supplementary Figure 9. Subgroup analysis of NCD indicators by age in Morocco** 15](#_Toc199146825)

[**Supplementary Figure 10. Subgroup analysis of NCD indicators by age in Zambia** 16](#_Toc199146826)

[**Supplementary Figure 11. Subgroup analysis of NCD indicators by age in Sri Lanka** 17](#_Toc199146827)

[**Supplementary Figure 12. Subgroup analysis of NCD indicators by age in Mumbai** 18](#_Toc199146828)

[**Supplementary Figure 13. Subgroup analysis of NCD indicators by education in Ecuador** 19](#_Toc199146829)

[**Supplementary Figure 14. Subgroup analysis of NCD indicators by education in Malawi** 20](#_Toc199146830)

[**Supplementary Figure 15. Subgroup analysis of NCD indicators by education in Morocco** 21](#_Toc199146831)

[**Supplementary Figure 16. Subgroup analysis of NCD indicators by education in Zambia** 22](#_Toc199146832)

[**Supplementary Figure 17. Subgroup analysis of NCD indicators by education in Sri Lanka** 23](#_Toc199146833)

[**Supplementary Figure 18. Subgroup analysis of NCD indicators by education in Mumbai** 24](#_Toc199146834)

1.

# Supplementary Table 1. Comparison of Socioeconomic Characteristics across four studied countries

| **Indicator** | **Ecuador** | **Malawi** | **Morocco** | **Zambia** | **India ^a^** | **Sri Lanka** |
| --- | --- | --- | --- | --- | --- | --- |
| Continent | America | Africa | Africa | Africa | Asia | Asia |
| Region | South America | Eastern Africa | Northern Africa | Eastern Africa | South Asia | Southeast Asian |
| WHO Region | Region of the Americas | African region | Eastern Mediterranean Region | African region | Asian region | Asian region |
| World Bank Income category | Upper-middle | Low | Lower-middle | Lower-middle ^c^ | Lower-middle | Lower-middle |
| Area | 106.889 mi^2^ | 45.747 mi^2^ | 172.414 mi^2^ | 290.585 mi^2^ | 604.3 km^2 a^ | 44.58 mi2 |
| Population (2020) | 17.64 million | 19.13 million | 36.9 million | 18.4 million | 18.4 million ^a^ | 21.92 million |
| Urban residents (2020) | 64.10% | 17.40% | 65.50% | 44.60% | 35% | 19.0 % |
| Gross Domestic Product (2020) | $98.1 billion | $12.18 billion | $114.73 billion | $18.1 billion | $2.623 billion | $80.71 billion |
| Gross National Income per capita (2020) | $5,530 | $580 | $3,020 | $1,160 | $1,900.71 | $3,666 |
| Median income (2022) | $3,525 | $484 | $2,716 | $545 | -- | $2,277 |
| Life expectancy at birth (in 2020) | 77y | 65y | 77y | 64y | 70y | 77y |
| Fertility rate (births per woman) | 2.4 | 4.1 | 2.4 | 4.5 | 2.2 | 2.2 |
| Under-five mortality rate (per 1000) | 13 | 39 | 19 | 61 | 33 | 7 |
| Coverage of immunization for measles | 83% | 92% | 99% | 93% | 89% | 96% |
| Literacy rate, population 15+ years (%) | 93 (2020) | 62 (2015) | 100 (2020) | 86.7 (2018) | 74.4 (2018) | 92.4 (2020) |
| Youth literacy rate, population 15-24 years, both sexes (%) | 98.8 (2020) | 72.9 (2015) | 97.7 (2018) | 92.1 (2018) | 91.7 (2018) | 98.9 (2020) |
| Elderly literacy rate, population 65+ years, both sexes (%) | 76.3 (2020) | 35.1 (2015) | 34.1 (2018) | 60.2 (2018) | 45.4 (2018) | 80.9 (2020) |
| Mobile subscriptions per 100 ^b^ | 88% | 48% | 128% | 80% | 84% | 139% |
| Global Gender Gap 2021 Ranking | 42 | 115 | 144 | 56 | 140 | 116 |
| Global Gender Gap 2021 Score | 0.739 | 0.671 | 0.612 | 0.726 | 0.625 | 0.670 |

^a^ Data specific for Mumbai

^b^ Data taken from ITU and corresponding to the year NCD-MPS was conducted

^c^ Was lower-middle income at time of survey. Has since moved to lower-income

# **Supplementary Table 2. Comparison of NCD-MPS design and implementation across studied countries**

| **Component** | **Ecuador** | **Malawi** | **Morocco** | **Zambia** | **Mumbai** | **Sri Lanka (T2)** |
| --- | --- | --- | --- | --- | --- | --- |
| **Date of fieldwork** | Jan. 31^st^ to Feb. 21^st^, 2020 | April 24^th^ to July 1^st^, 2019 | Jan. 28^th^ to Feb.28^th^, 2019 | July 27^th^ to Oct. 14^th^, 2017 | Nov. 15^th^, 2021, to Jan 21^st^, 2022 | Nov. 1^st^ to Dec. 6^th^, 2021, and  Dec. 6^th^, 2021, to Jan. 21^st^, 2022 |
| **Sampling** | A two-phase sample of mobile phone numbers generated via RDD, using the mobile phone prefixes for CNT, Claro, and Movistar stratified by age and sex in the second phase. | A two-phase sample of mobile phone numbers generated via RDD using the mobile phone prefixes for Airtel and TNM stratified by age and sex in the second phase. | A two-phase sample of mobile phone numbers generated via RDD using the mobile phone prefixes for Maroc Telecom, INWI, & Orange stratified by age and sex in the second phase. | A two-phase sample of mobile phone numbers generated via RDD using the mobile phone prefixes for Airtel, MTN and Zamtel, stratified by age and sex in the second phase. | A two-phase sample of mobile phone numbers generated via RDD using the mobile phone prefixes for Airtel, MTNL, Reliance Jio, and Vodafone Idea stratified by age and sex in the second phase. | A two-phase sample of mobile phone numbers generated via RDD using the mobile phone prefixes for Dialog, Mobitel, Etisalat/Hutch, and Airtel. Sri Lanka stratified by age and sex in the second phase. |
| **Number of**  **Interviews** | 3,101 interviews, allocated proportionally across strata to general population distribution. | 5,814 interviews, allocated proportionally across strata to general population distribution. | 3,515 interviews, allocated proportionally across strata to general population distribution. | 6,056 interviews, allocated proportionally across strata to general population distribution. | 3,418 interviews, allocated proportionally across strata to general population distribution. | 4,356 interviews, allocated proportionally across strata to general population distribution. |
| **Strata** | 6 strata, by crossing sex (male, female) with age (18-29, 30-44, 45-69). | 6 strata, by crossing sex (male, female) with age (18-29, 30-44, 45-69) | 6 strata, by crossing sex (male, female) with age (18-29, 30-44, 45-69) | 6 strata, by crossing sex (male, female) with age (18-29, 30-44, 45-69) | 6 strata, by crossing sex (male, female) with age (18-29, 30-44, 45-69) | 6 strata, by crossing sex (male, female) with age (18-29, 30-44, 45-69) |
| **Questionnaire** | 24 questions; administered in Spanish. | 18 questions; administered in three languages (English, Nyanja, Tambuka). | 20 questions; administered in two languages (Moroccan Arabic and French). | 20 questions; 8 languages: English, Bemba, Nyanja, Tonga, Luvale, Lozi, Kaonde, Lunda | 20 questions in 3 languages: Marathi, Hindi, and English | 24 core questions 3 languages: Tamil, Sinhala, English |
| **Contact times** | 7 days of the week,  from 9am-8pm | 7 days of the week,  from 8am-8pm | 7 days of the week,  from 8am-8pm | 7 days of the week,  from 8am-8pm | 7 days of the week,  from 8am-8pm | 7 days of the week,  from 8am-8pm |
| **Contact attempts** | Contact #1: IVR  #2: IVR, 26 hours after #1  #3: IVR, 26 hours after Contact #2 | Contact #1: IVR;  #2: IVR, 50 hours after #1;  #3: SMS, 50 hours after #2;  #4: SMS, 50 hours after #3. | Contact #1: IVR;  #2: IVR, 50 hours after #1;  #3: SMS, 50 hours after #2;  #4: SMS, 50 hours after #3 | Contact #1: IVR;  #2: IVR, 50 hours after #1;  #3: SMS, 50 hours after #2;  #4: SMS, 50 hours after #3 | Contact #1: IVR  #2: IVR, 26 hours after #1  #3: IVR, 26 hours after #2 | Contact #1: IVR  #2: IVR, 50 hours after #1  #3: IVR, 50 hours after #2 |
| **Cost to**  **Respondents** | None. | None | None | None | None | None |
| **Incentives** | $1 USD. | 720 Kwacha credit ($0.88 USD). | 10 Dirham credit ($0.99 USD). | 10 Kwacha credit ($0.59 USD). | 75 Rupees credit | None |
| **Tool and Hosting** | Surveda; data hosted at Ecuador Ministry of Health | Surveda; data hosted at Malawi Ministry of Health | Surveda; data hosted at Morocco Ministry of Health | Surveda; data hosted at Zambia National Data Center | Surveda; data hosted at Municipal Corporation of Greater Mumbai | Surveda; with data hosted at the Sri Lanka Ministry of Health |
| **Mobile Phone Subscribers per 100^1^** | 87.8 per-100  (2020) | 47.8 per 100  (2019) | 128.0 per 100  (2019) | 79.7 per 100  (2017) | 84 per 100  (2020) | 139 per 100  (2020) |
| **Estimated Total Mobile Phone Subscriptions** | 15.5 million  (2020) | 8.9 million  (2019) | 46.7 million  (2019) | 13.1 million  (2017) | 17.1 million  (2020) | 30.5 million  (2020) |
| **Response Rate (RR6)** | 9.9% | 7.0% | 8.3% | 6.3% | 3.1% | 7.6% |

^1^Number of subscriptions to a public mobile-telephone service that provides access to the public switched telephone network using cellular technology. The indicator includes (and is split into) the number of postpaid subscriptions and the number of active prepaid accounts) /population x 100 population. Sources: Agencia de Regulación y Control de las Telecomunicaciones (Ecuador); Malawi Communication regulatory authority (Malawi); The National Telecommunications Regulatory Agency (Morocco); Zambia Information & Communications Technology Authority (Zambia). Note: ITU (International Communication Union) and World Bank’s report use these same sources

# **Supplementary Table 3. Comparison of WHO STEPS design and implementation across studied countries**

| **Component** | **Ecuador** | **Malawi** | **Morocco** | **Zambia** | **Mumbai** | **Sri Lanka** |
| --- | --- | --- | --- | --- | --- | --- |
| **Date of fieldwork** | May-June 2018 ^a^ | Oct-Nov 2017 ^a^ | Mar 1^st^ -May 28^th^, 2017 | July 22^nd^-Oct. 15^th^, 2017 | 2021 | 2021 |
| **Sampling** | Obtained through a probabilistic multi-stage cluster sampling method of three stages: 1) PSU selection by strata; 2) selection of 12 inhabited households (16 later in the process) within each PMU; 3) selection of one person aged 18-69 per household. Poststratification was used to correct sampling weights. Estimated sample size: 6680. | Obtained through a probabilistic multi-stage sampling method of three stages: 1) EA (PSU) selection by means of the PPS sampling method; 2) selection of 20 households within each EA; 3) selection of one person aged 18-69 per household. Sampling weights were used to enable representativeness of target population. Estimated sample size: 5088. | Obtained through a probabilistic multi-stage sampling method of four stages: 1) PSU selection; 2) SSU selection; 3) selection of 25 households within each SSU; 4) selection of one-person aged 18+ per household. Sampling weights were used to enable representativeness of target population. Estimated sample size: 6100. | Obtained through a probabilistic multi-stage cluster sampling method of three stages: 1) SEA (PSU) selection by means of the PPS sampling method; 2) selection of households within urban (n=20) and rural (n=15) SEAs; 3) selection of one person aged 18-69 per household. Sampling weights were used to enable representativeness of target population. Estimated sample size: 5791. | Obtained through a probabilistic multi-stage cluster sampling method of three stages: 1) 238 Census Blocks (PSU) selection by means of the PPS sampling method; 2) selection of 25 households per PSU through systematic random sampling; 3) selection of one person aged 18-69 per household. Sampling weights were used to enable representativeness of target population. Estimated sample size: 5950. | Obtained through a probabilistic multi-stage cluster sampling method of three stages: 1) 664 PSUs (Census Blocks) selected using PPS sampling; 2) selection of 12 households per PSU; and 3) selection of one eligible person aged 18-69 per household. Sampling weights were used to enable representativeness of target population. Estimated sample size: 7228. |
| **Sampling Frame** | Population and Housing Census, 2010 (Censo de Población y Vivienda, 2010). | List of EA (third level of administrative division) provided by the National Statistics Office (NSO). | The master sample from 2014, drawn by the Higher Planning Commission from the Population and Housing Census from the same year. | Household listing from the Zambia Population-  Based HIV Impact Assessment (ZAMPHIA). | Household listing and mapping exercise done just prior to field work in selected PSUs. | Census of Population and Housing – 2011, which is updated for the third quarter Labor Force Survey in 2020 |
| **Number of**  **Interviews** | 4638 | 4187 | 5429 | 4302 | 5199 | 6267 |
| **Strata** | 2 strata, created by crossing sex (male, female) with age (18-69). | 8 strata, created by crossing sex (male, female) with age (18-29, 30-44, 45-59, 60-69) | 10 strata, created by crossing sex (male, female) with age (18-29, 30-44, 45-59, 60-69, 70+) | 8 strata, created by crossing sex (male, female) with age (18-29, 30-44, 45-59, 60-69) | 8 strata, created by crossing sex (male, female) with age (18-29, 30-44, 45-59, 60-69) | 8 strata, created by crossing sex (male, female) with age (18-29, 30-44, 45-59, 60-69) |
| **Questionnaire** | WHO STEPS standard questionnaire (V3.2), translated to Spanish, and adapted to the Ecuadorian context. | WHO STEPS standard questionnaire (V3.2), aside from English version, translated to Chichewa and Tumbuka. | WHO STEPS standard questionnaire (V3.1), which was adapted and translated to Arabic and French. | WHO STEPS standard questionnaire (V3.2), aside from English, translated to the seven main local languages. | WHO STEPS standard questionnaire (V3.2), which was adapted and translated to Hindi and Marathi. | WHO STEPS standard questionnaire (V3.2), which was adapted and translated to Sinhala and Tamil. |
| **Measurement Duration** | Complete survey lasted two days: WHO STEPS 1 and 2 were conducted on day 1; and Step 3, on day 2. | Complete survey lasted two days: WHO STEPS 1 and 2 were conducted on day 1; and Step 3, on day 2 | Complete survey lasted two days: WHO STEPS 1 and 2 were conducted on day 1; and Step 3, on day 2 | Complete survey lasted two days: WHO STEPS 1 and 2 were conducted on day 1; and Step 3, on day 2. Some participants had Step 3 done on same day. | Complete survey lasted two days: WHO STEPS 1 and 2 were conducted on day 1; and Step 3, on day 2. | Complete survey lasted two days: WHO STEPS 1 and 2 were conducted on day 1; and Step 3, on day 2. |
| **Contact Attempts** | Not indicated in the official report. | Not indicated in the official report. | Three visits in different moments, distributed in two days. | Two follow-up visits once individual is selected for the household. | At least one follow-up attempt. | One follow-up attempt. |
| **Tools and Hosting** | Android tablets with eWHO STEPS and ODK app; hosted by ona.io. | Tablets with eWHO STEPS and ODK app; uploaded to a central server hosted by ona.io. | Tablets with eWHO STEPS; hosted at a server located at the Ministry of Health. | Handheld Android devices with eWHO STEPS; hosted at Centre for infectious Diseases Research | Android tablets using eWHO STEPS and ODK app; hosted by ona.io. | Tablets with eWHO STEPS software; hosted at a server located at the Ministry of Health. |
| **Response Rate** | 69.4% | 82.3% | 89.0% | 74.3% | 87% | 81% |

PSU: Primary Sampling Unit; SSU: Secondary Sampling Unit; EA: Enumeration Areas; SEA: Standard Enumeration Areas; PPS: Probability Proportional to Size; DS: Divisional Secretariat; ^a^ Exact fieldwork dates not indicated in the official report.

# **Supplementary Table 4. Sociodemographic characteristics of NCD-MPS and WHO STEPS participants across countries and the city of Mumbai (unweighted estimates)**

|  | **Ecuador** | | **Sri Lanka** | | **Morocco** | | **Zambia** | | **Mumbai** | | **Malawi** | |
| --- | --- | --- | --- | --- | --- | --- | --- | --- | --- | --- | --- | --- |
|  | **MPS** | **STEPS** | **MPS** | **STEPS** | **MPS** | **STEPS** | **MPS** | **STEPS** | **MPS** | **STEPS** | **MPS** | **STEPS** |
| **Sex** |  |  |  |  |  |  |  |  |  |  |  |  |
| Male | 49.6 (1518) | 41.9 (1944) | 53.9 (2298) | 39.0 (2447) | 53.0 (1788) | 34.8 (1708) | 54.9 (3237) | 37.5 (1614) | 61.0 (2014) | 50.0 (2601) | 64.4 (3690) | 35.5 (1485) |
| Female | 50.4 (1543) | 58.1 (2694) | 46.1 (1964) | 61.0 (3820) | 47.0 (1587) | 65.2 (3283) | 45.1 (2654) | 62.5 (2688) | 39.0 (1286) | 50.0 (2598) | 35.6 (2044) | 64.5 (2702) |
| **Age (years)** |  |  |  |  |  |  |  |  |  |  |  |  |
| 18-29 | 33.9 (1038) | 26.0 (1205) | 27.6 (1178) | 16.0 (1002) | 43.6 (1472) | 24.7 (1061) | 50.5 (2976) | 37.7 (1620) | 37.5 (1236) | 26.8 (1391) | 54.5 (3124) | 32.7 (1371) |
| 30-44 | 34.3 (1049) | 34.4 (1596) | 35.3 (1505) | 33.3 (2086) | 33.7 (1137) | 40.5 (1744) | 34.6 (2040) | 35.2 (1513) | 36.5 (1205) | 42.7 (2222) | 34.9 (2004) | 37.0 (1548) |
| 45-69 | 31.8 (974) | 39.6 (1837) | 37.0 (1579) | 50.7 (3179) | 22.7 (766) | 34.8 (2186) | 14.9 (875) | 27.2 (1169) | 26.0 (859) | 30.5 (1586) | 10.6 (606) | 30.3 (1268) |
| **Education** |  |  |  |  |  |  |  |  |  |  |  |  |
| Up to primary | 21.4 (647) | 52.9 (2452) | 4.2 (175) | 15.8 (991) | 28.6 (944) | 71.0 (3437) | 20.3 (1226) | 60.1 (2582) | 39.9 (1299) | 24.1 (1242) | 23.1 (1305) | 45.4 (1899) |
| More than primary to up to secondary | 44.0 (1330) | 33.2 (1538) | 37.2 (1574) | 61.8 (3868) | 41.7 (1377) | 26.9 (1136) | 35.5 (2139) | 32.3 (1390) | 16.0 (521) | 34.7 (1786) | 56.7 (3200) | 51.1 (2136) |
| More than secondary | 34.6 (1084) | 13.9 (644) | 58.7 (2485) | 22.5 (1405) | 29.8 (984) | 7.8 (413) | 41.3 (2485) | 7.6 (327) | 44.1 (1433) | 41.2 (2121) | 20.2 (1141) | 3.5 (148) |

Data are % with absolute frequencies between brackets

# **Supplementary Table 5. Final disposition case codes and outcome rates for dialed phone numbers in mobile phone survey across countries and the city of Mumbai**

| **Disposition** | **Ecuador**  **n= 50,740** | | **Sri Lanka**  **n= 131,140** | | **Morocco**  **n= 326,359** | **Zambia**  **n= 339,073** | **Mumbai**  **n= 401,278** | **Malawi**  **n= 238,569** |
| --- | --- | --- | --- | --- | --- | --- | --- | --- |
| Complete Interview | 2254 (4.5%) | | 2868 (2.2%) | | 1454 (0.5%) | 3397 (1.0%) | 2102 (0.5%) | 3693 (1.6%) |
| Partial Interview | 847 (1.7%) | | 1498 (1.1%) | | 2061 (0.6%) | 2659 (0.8%) | 1316 (0.3%) | 2121 (0.9%) |
| Breakoff: Eligible | 233 (0.5%) | | 3878 (3.0%) | | 389 (0.1%) | 450 (0.1%) | 5337 (1.3%) | 494 (0.2%) |
| Ineligible: Underage | 466 (0.9%) | | 2144 (1.6%) | | 69 (0.02%) | 1816 (0.5%) | 2068 (0.5%) | 2504 (1.1%) |
| Ineligible: Quotas | 1593 (3.1%) | | 11892 (9.1%) | | 5057 (1.6%) | 4688 (1.4%) | 7538 (1.9%) | 5074 (2.1%) |
| Ineligible: Non-resident |  | |  | |  |  | 13663 (3.4%) |  |
| Refused | 3578 (7.1%) | | 39083 (29.8%) | | 253 (0.08%) | 7341 (2.2%) | 26215 (6.5%) | 2786 (1.2%) |
| Breakoff: unknown eligibility | 1030 (2.5%) | | 4642 (3.5%) | | 20298 (6.8%) | 12035 (3.6%) | 26634 (6.6%) | 12936 (6.2%) |
| No answer | 40739 (80.3%) | | 65135 (49.7%) | | 296778 (90.9%) | 306687 (90.5%) | 316405 (78.8%) | 208962 (87.6%) |
| **Survey outcome rates** |  |  | |  |  |  |  |  |
| Contact Rate 3 | 6912/47651 (14.5%) |  | | 47327/112462 (42.1%) | 4157/300935 (1.4%) | 13847/320534 (4.4%) | 34970/351375 (10.0%) | 9094/218056 (4.2%) |
| Refusal Rate 3 | 3811/47651 (8.0%) |  | | 42961/112462 (38.2%) | 642/300935 (0.21%) | 7791/320534 (2.4%) | 31546/351375 (9.0%) | 3280/218056 (1.5%) |
| Cooperation rate 3 | 3101/6912 (44.9%) |  | | 4366/47327 (9.2%) | 3515/4157 (84.6%) | 6056/13847 (43.7%) | 3418/34970 (9.8%) | 5914/9194 (64.3%) |
| Response Rate 6 * | 9.9% |  | | 9.0% | 8.3% | 6.3% | 3.1% | 7.0% |

Data are n (%) or n/N (%). Contact rate #3, Refusal rate #3, Cooperation rate #3, and Response rate #6 were calculated using the standard definitions proposed by the American Association for Public Opinion Research. * Adjusted by design. The sampling design involved two Phases. Each phase had a response rate. The final response rate was the product of Phase I and Phase II response rates.

# **Supplementary Figure 1. Subgroup analysis of NCD indicators by sex in Ecuador**

**
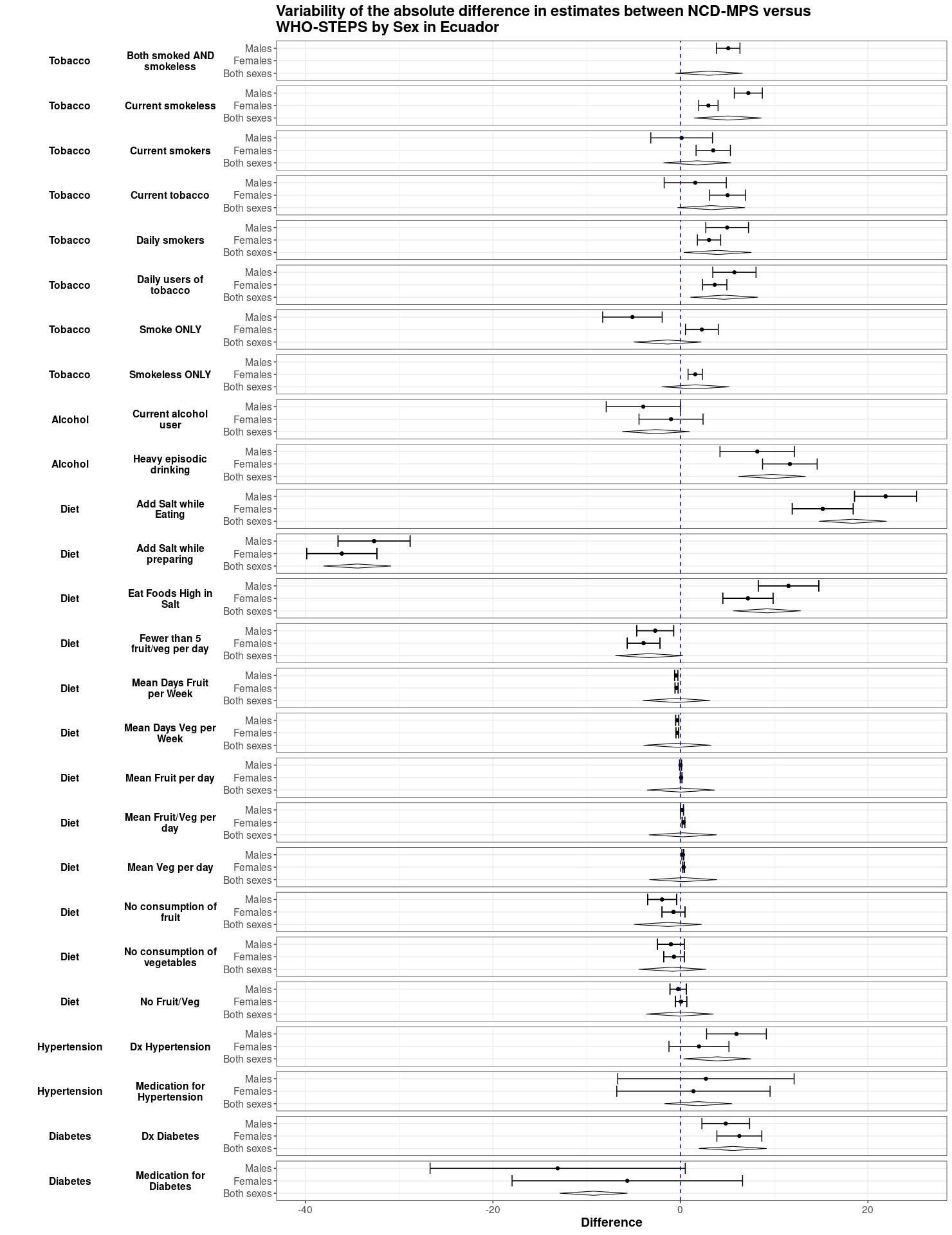
**

# **Supplementary Figure 2. Subgroup analysis of NCD indicators by sex in Malawi**


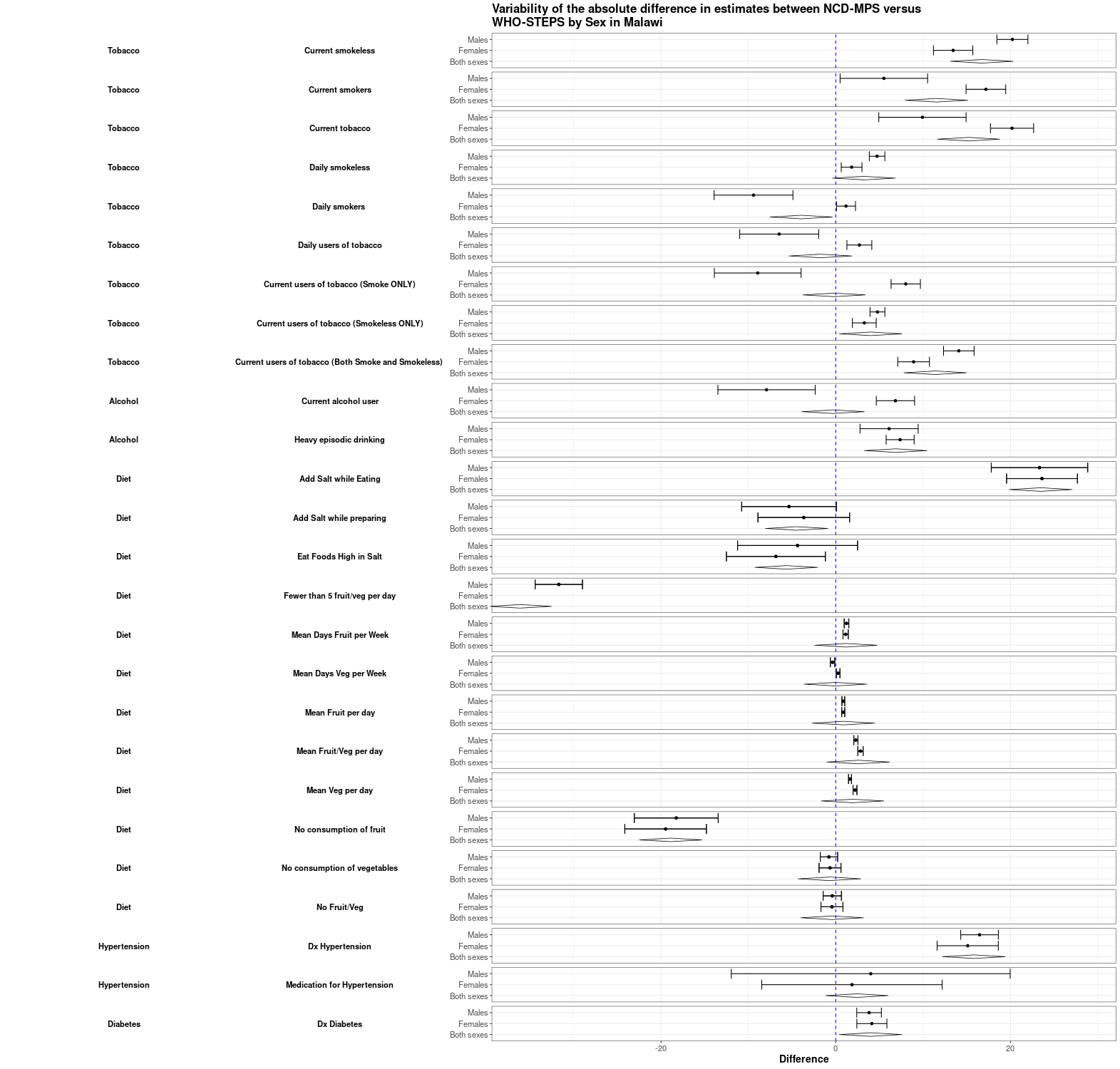


# **Supplementary Figure 3. Subgroup analysis of NCD indicators by sex in Morocco**


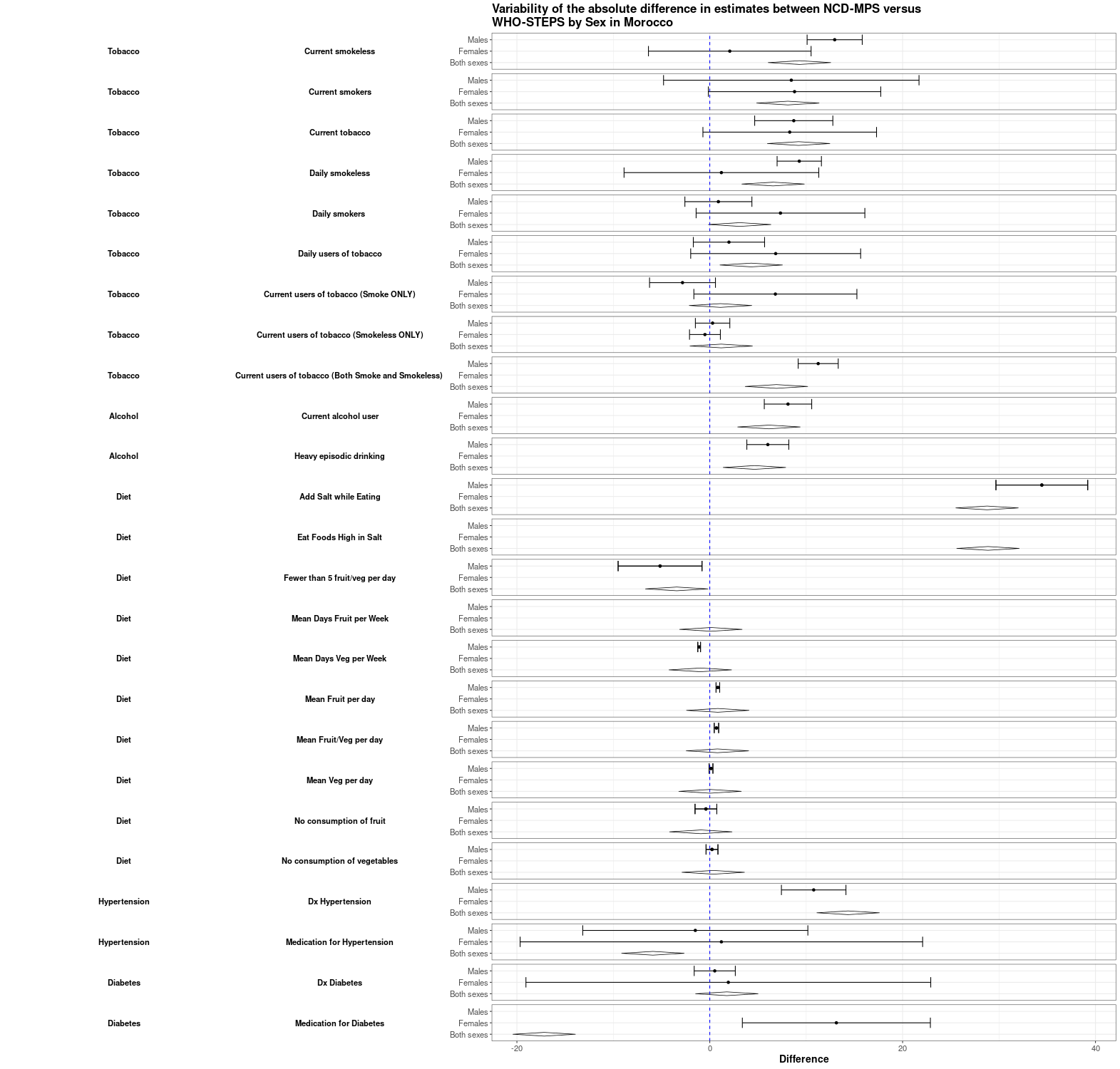


# **Supplementary Figure 4. Subgroup analysis of NCD indicators by sex in Zambia**

**
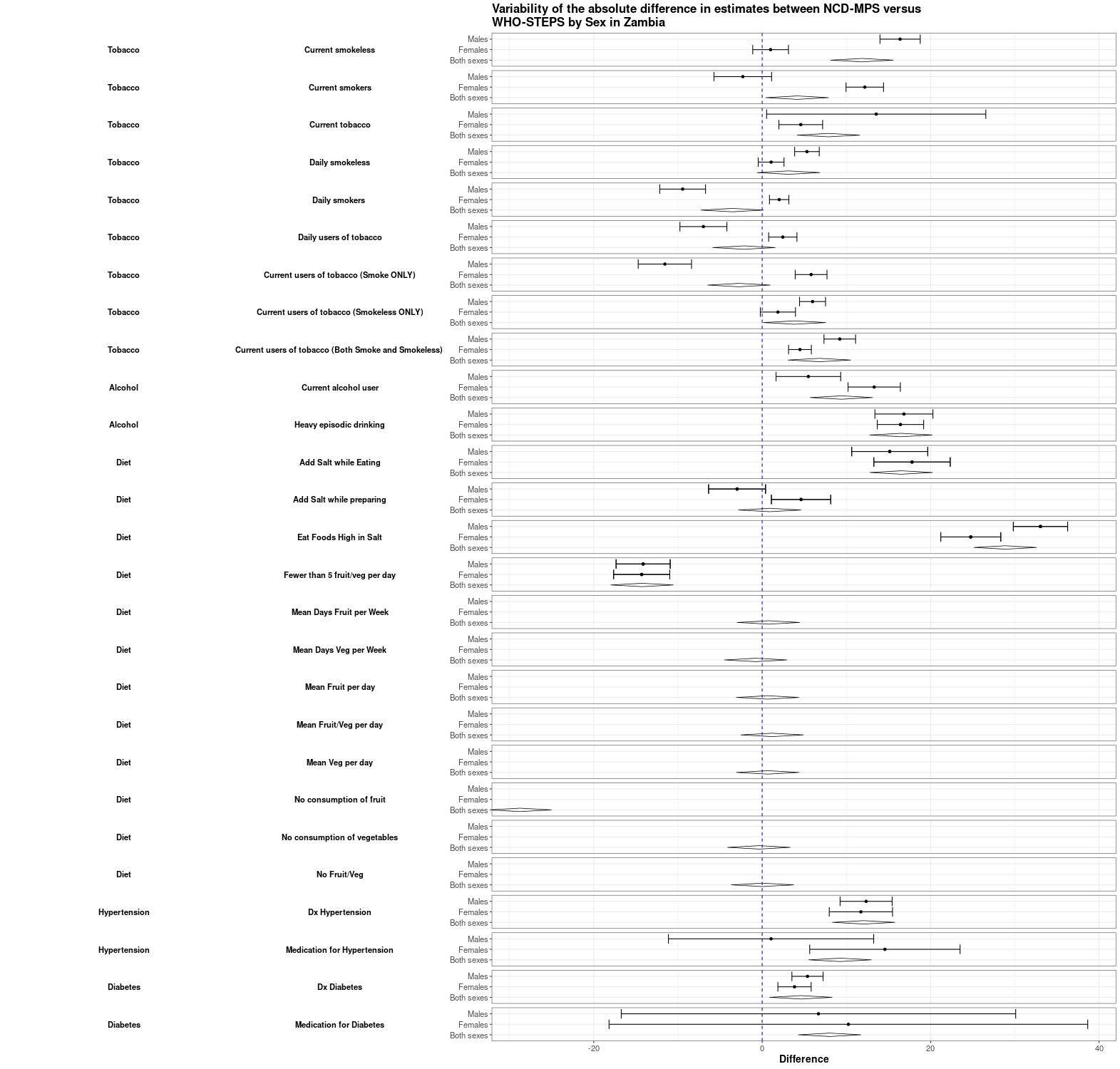
**

# **Supplementary Figure 5. Subgroup analysis of NCD indicators by sex in Sri Lanka**

**
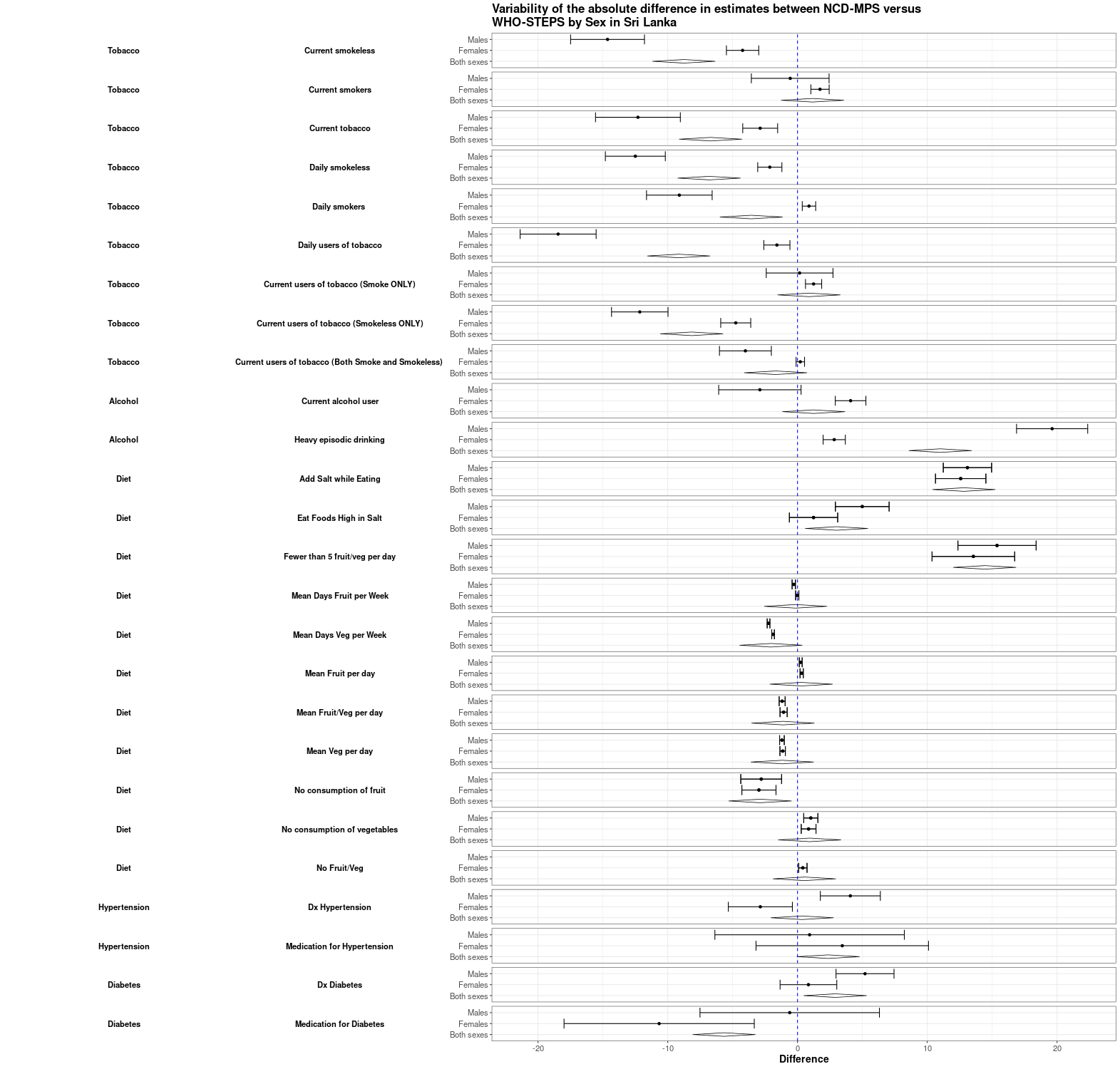
**

# **Supplementary Figure 6. Subgroup analysis of NCD indicators by sex in Mumbai**

**
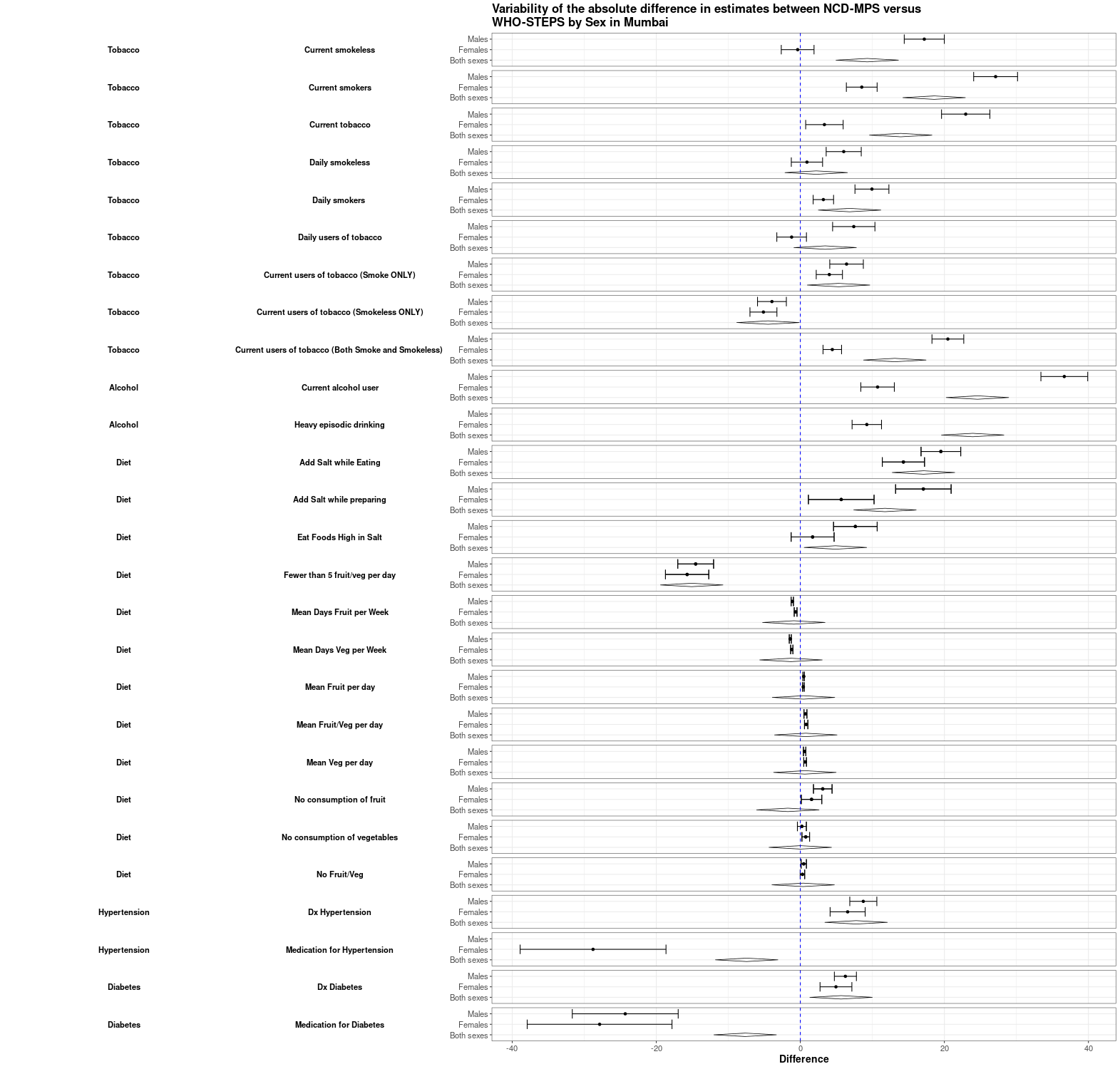
**

# **Supplementary Figure 7. Subgroup analysis of NCD indicators by age in Ecuador**

**
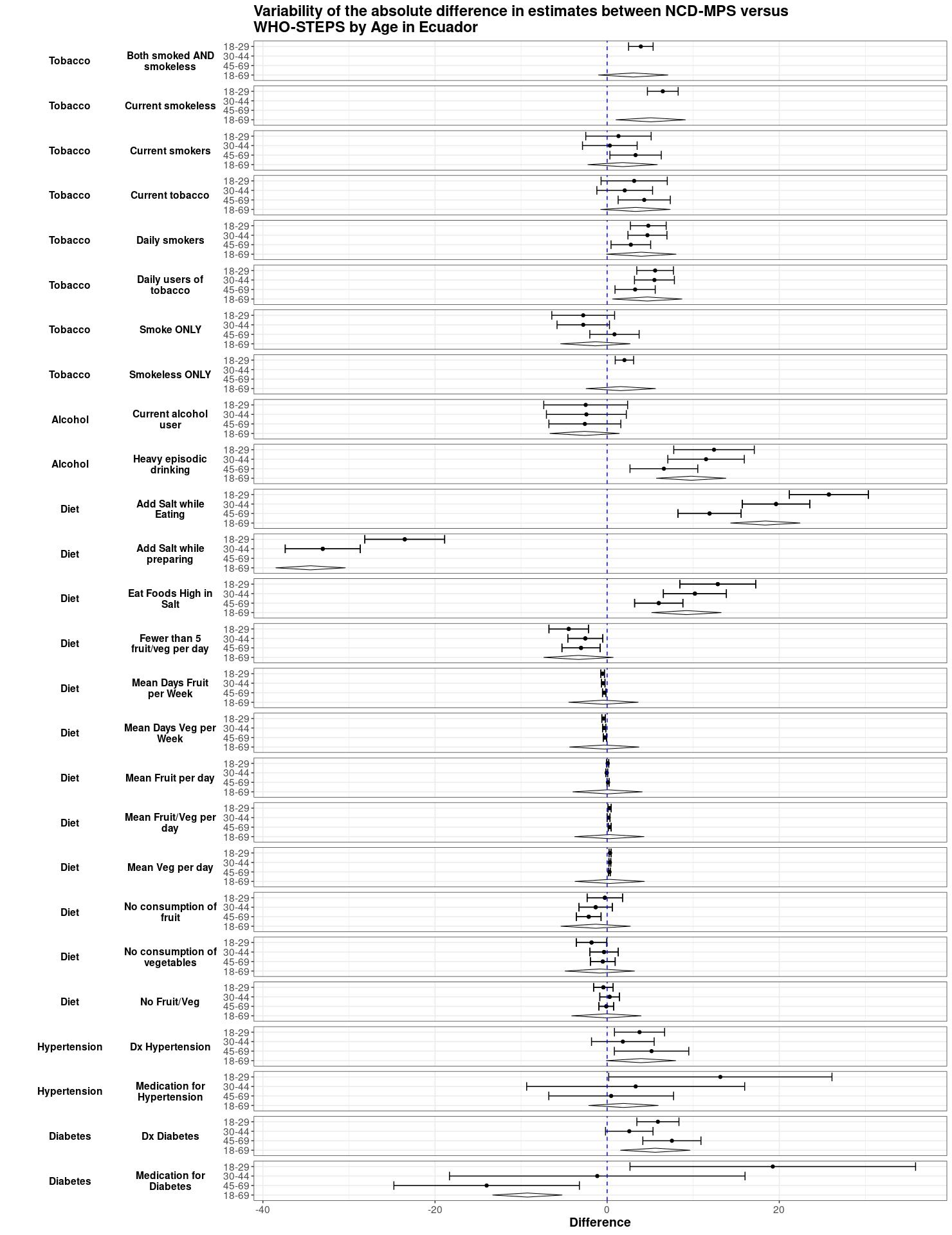
**

# **Supplementary Figure 8. Subgroup analysis of NCD indicators by age in Malawi**

**
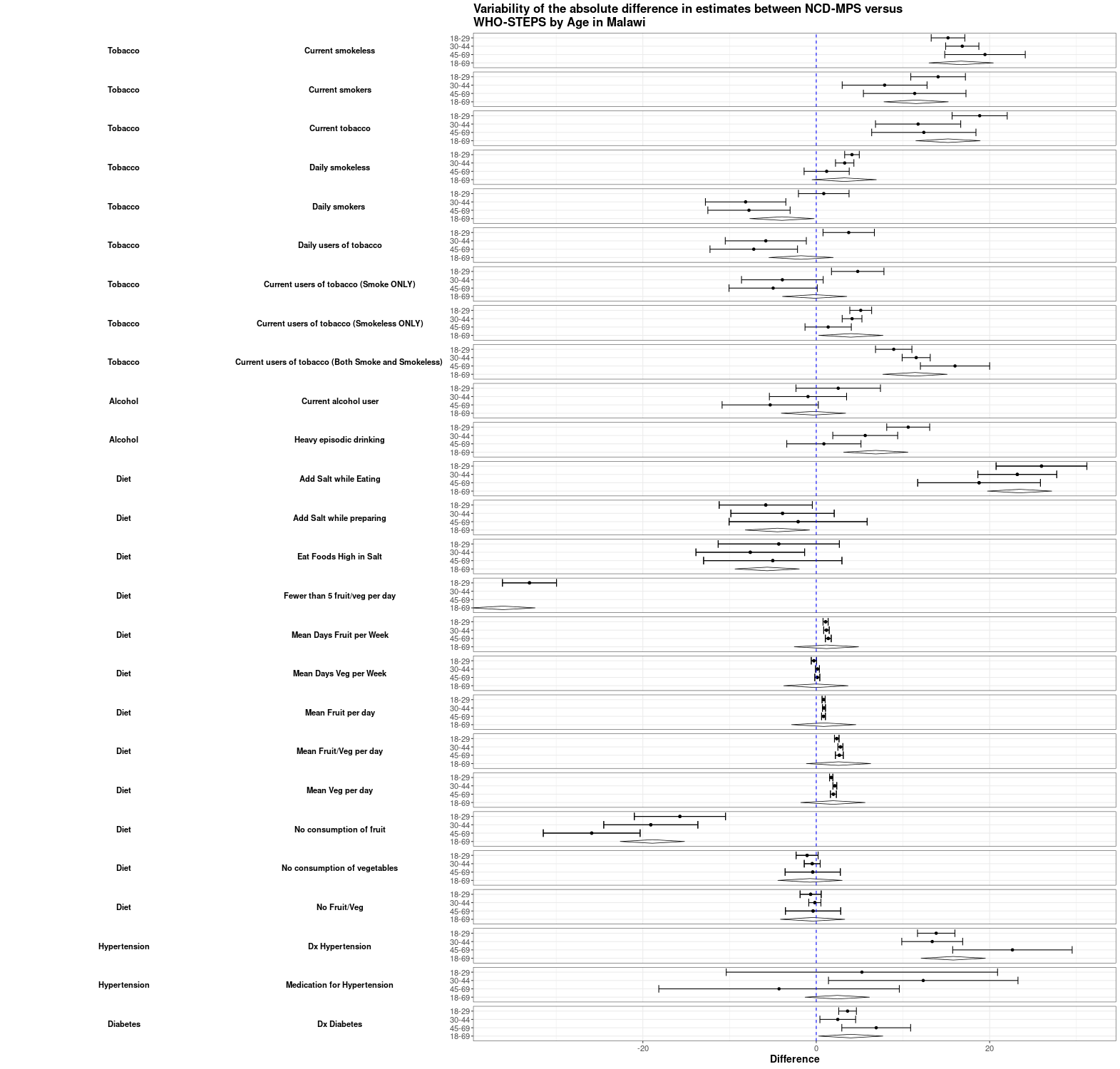
**

# **Supplementary Figure 9. Subgroup analysis of NCD indicators by age in Morocco**

**
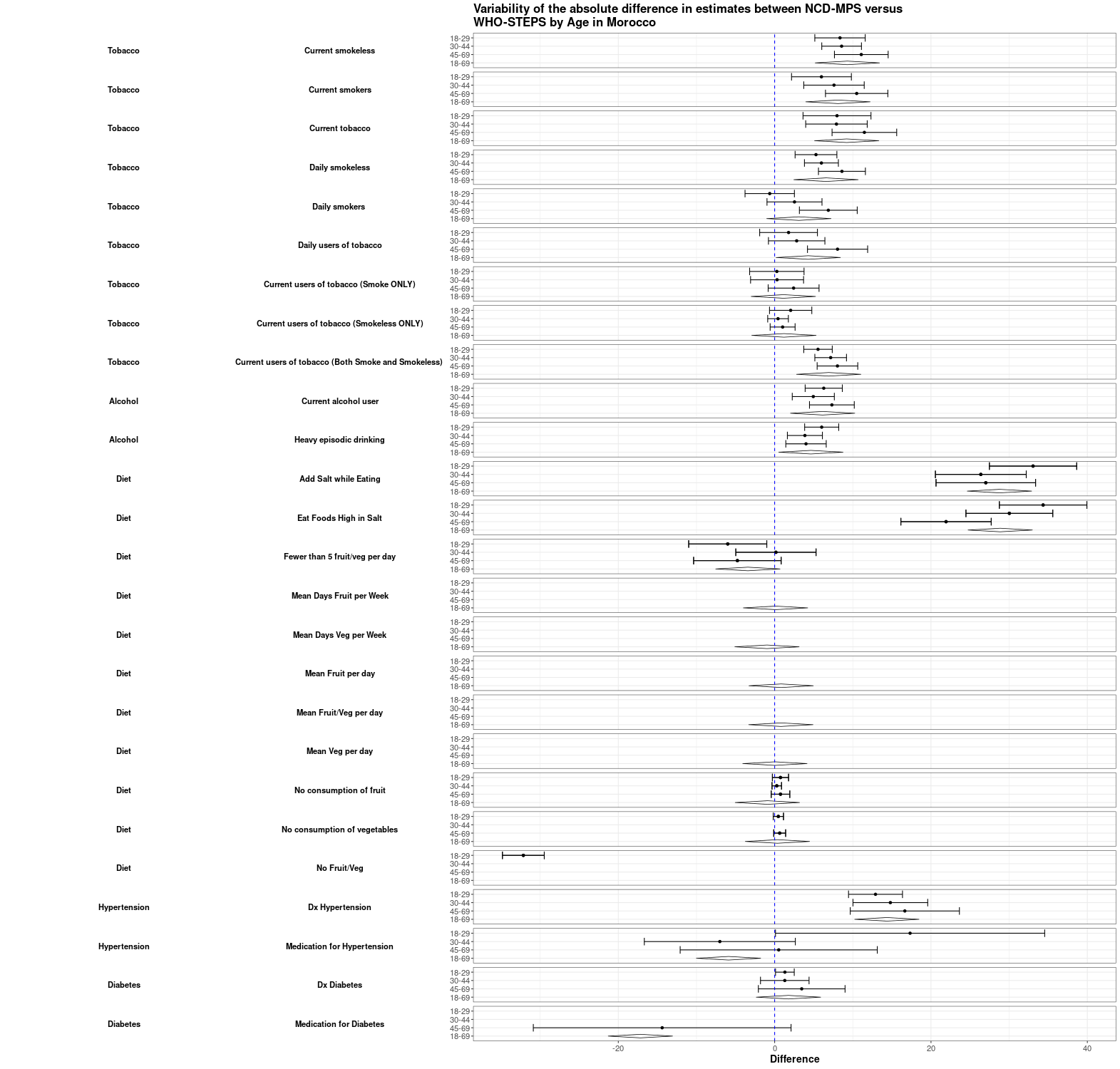
**

# **Supplementary Figure 10. Subgroup analysis of NCD indicators by age in Zambia**

**
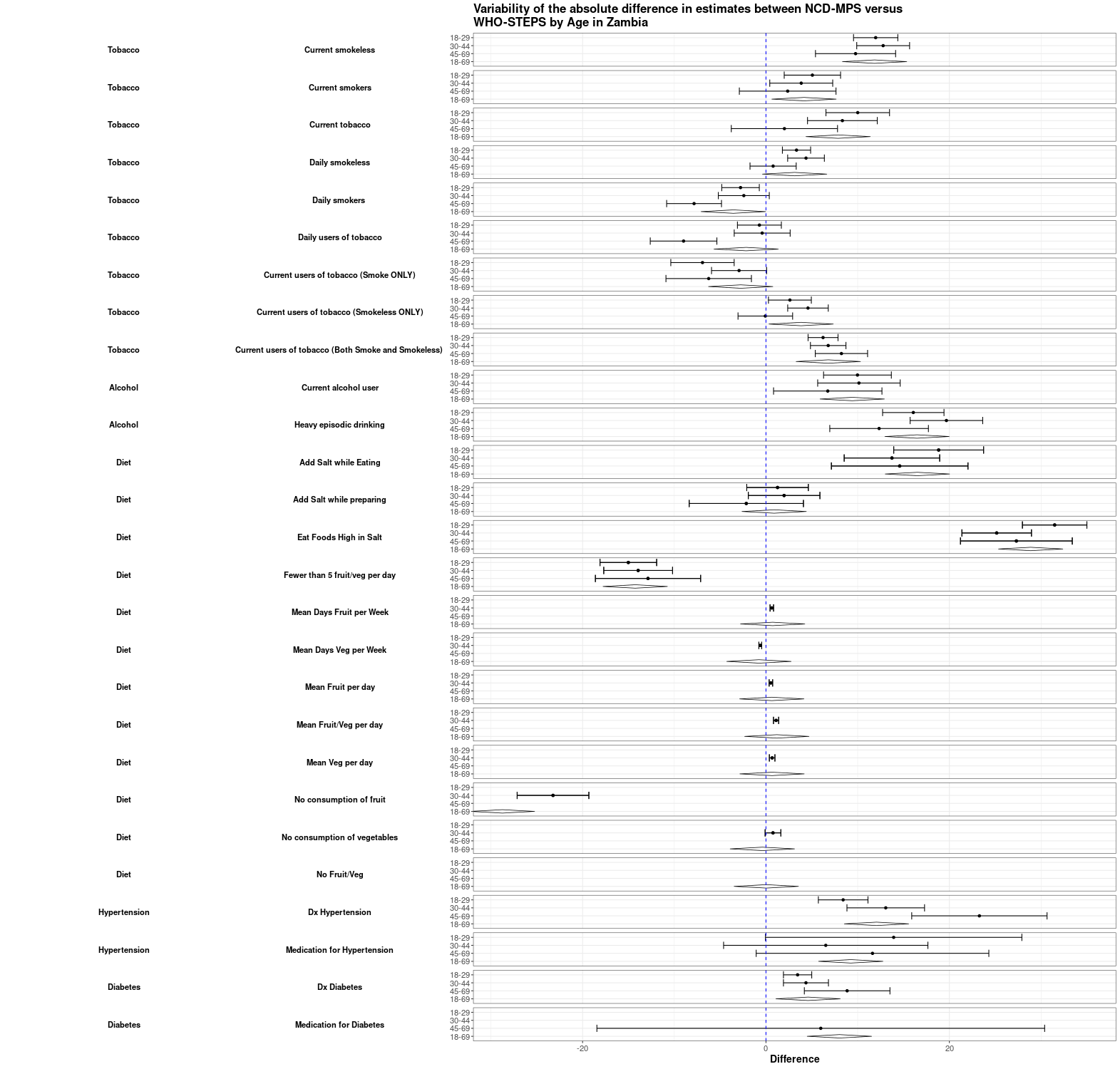
**

# **Supplementary Figure 11. Subgroup analysis of NCD indicators by age in Sri Lanka**


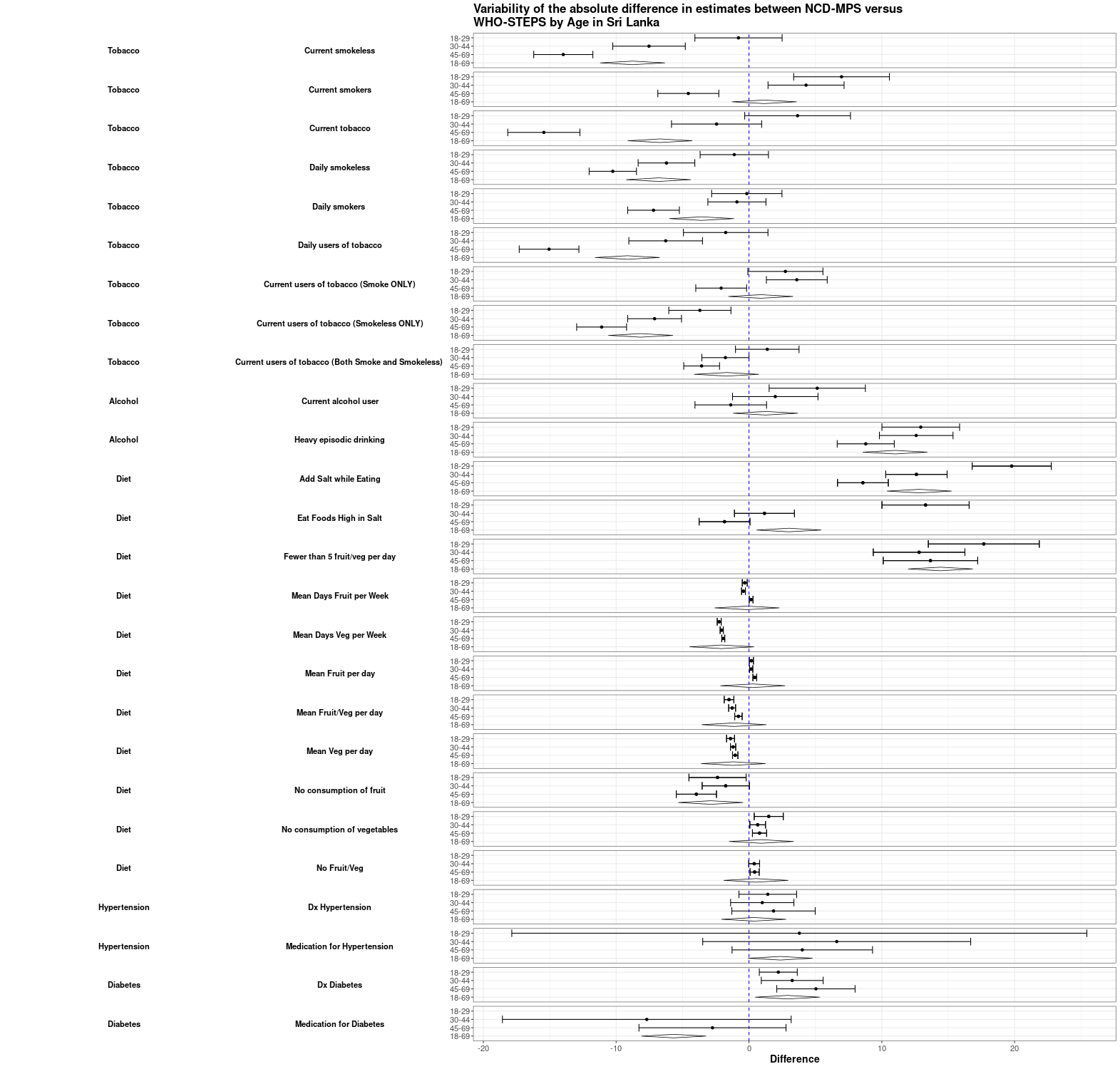


# **Supplementary Figure 12. Subgroup analysis of NCD indicators by age in Mumbai**


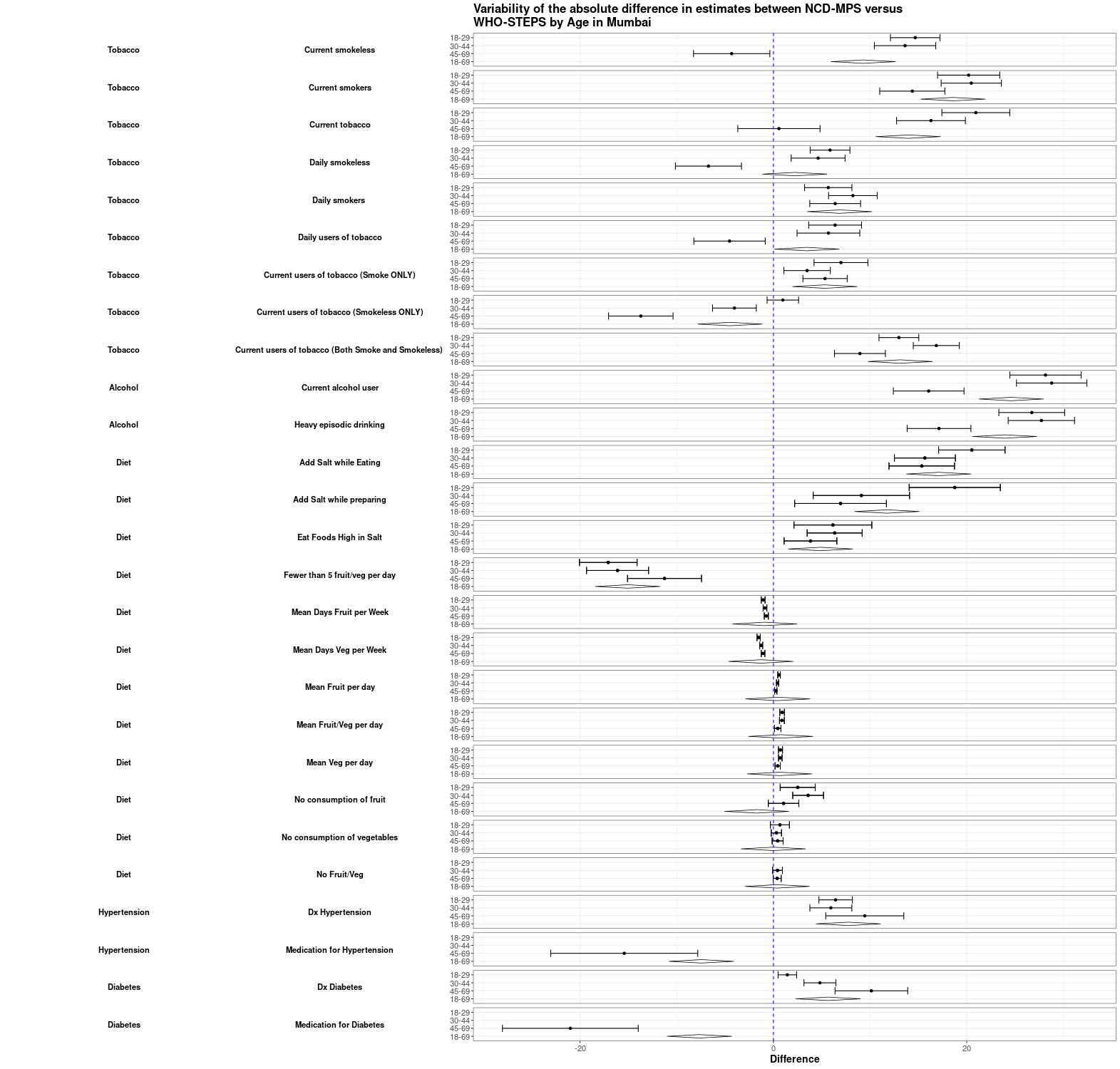


# **Supplementary Figure 13. Subgroup analysis of NCD indicators by education in Ecuador**

**
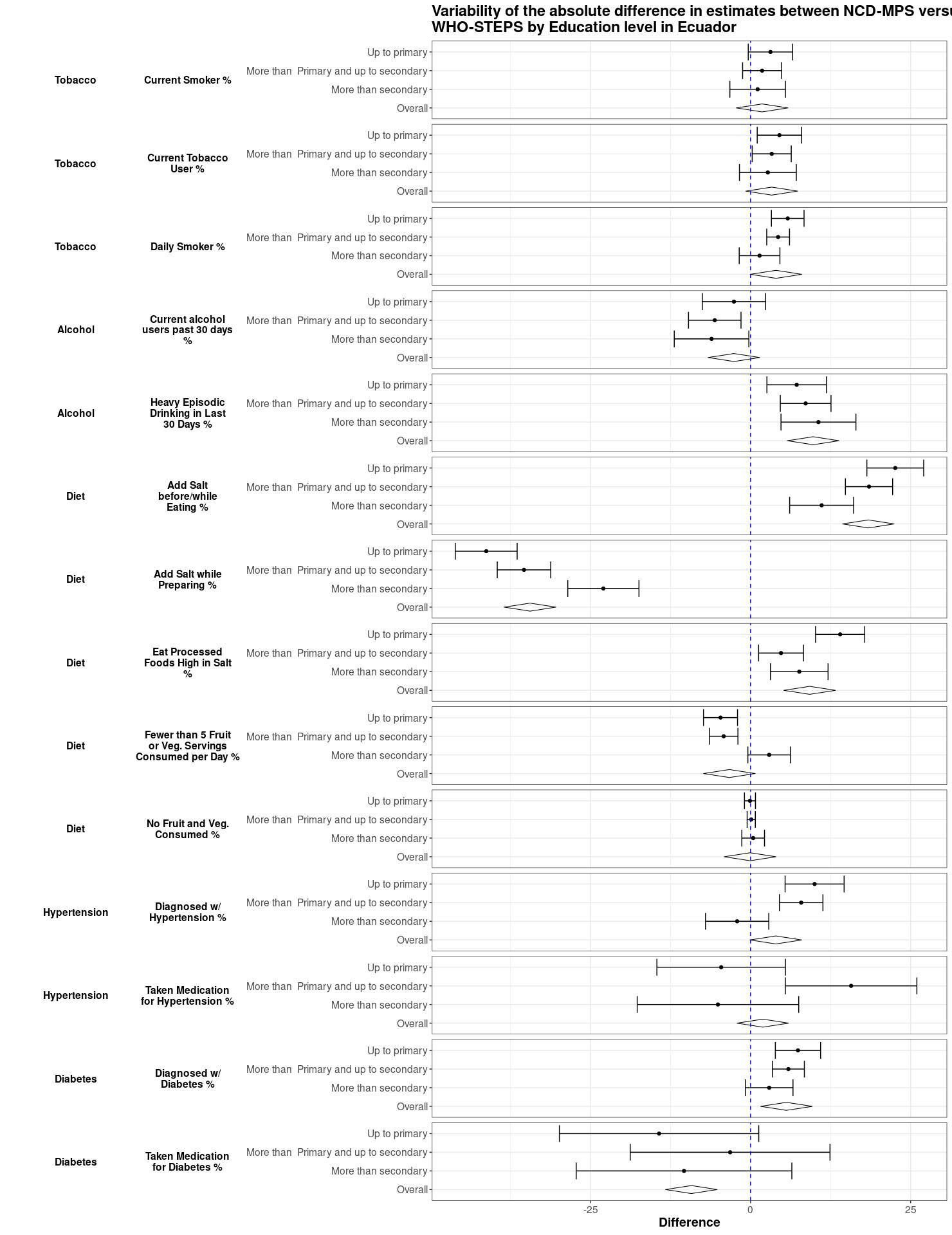
**

# **Supplementary Figure 14. Subgroup analysis of NCD indicators by education in Malawi**


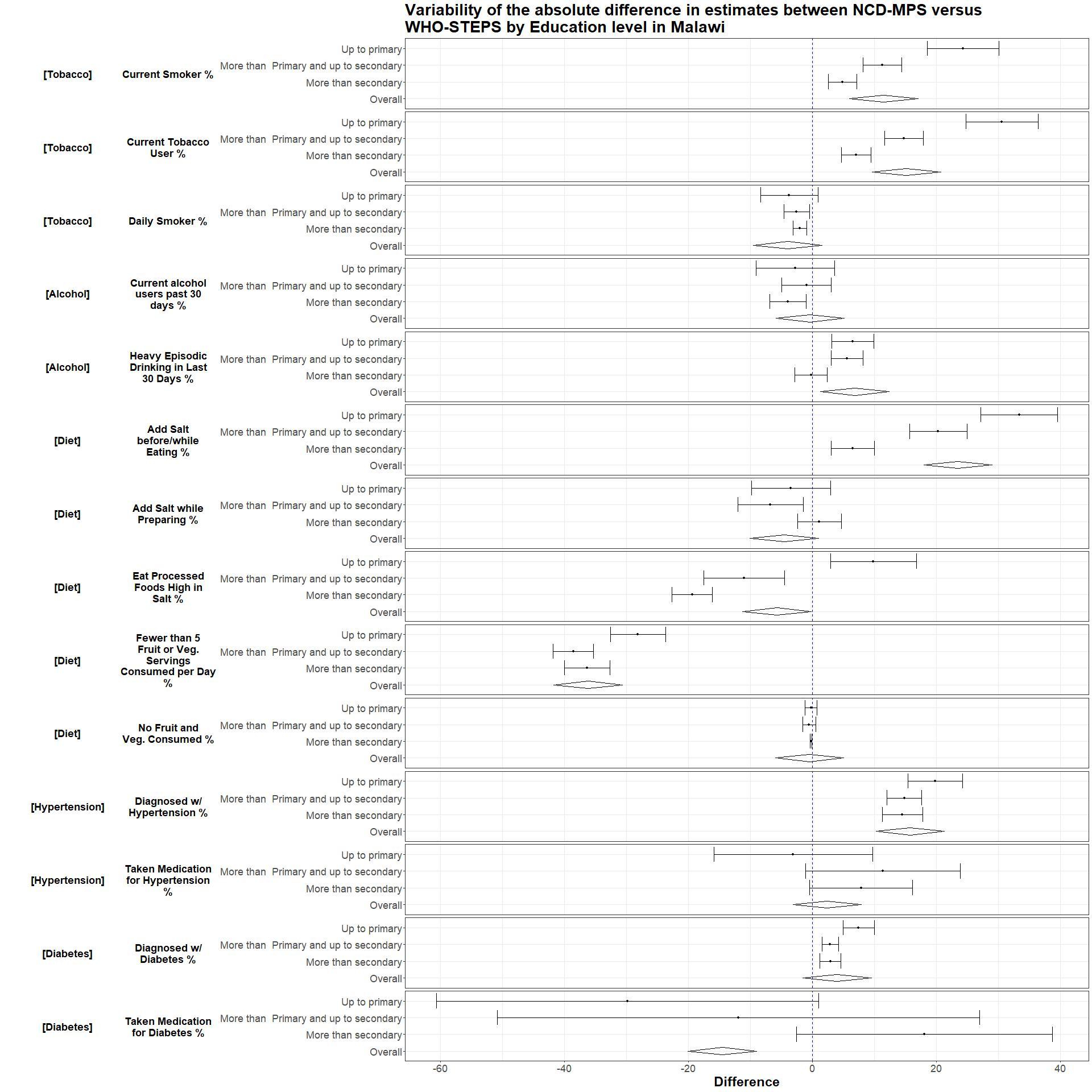


# **Supplementary Figure 15. Subgroup analysis of NCD indicators by education in Morocco**


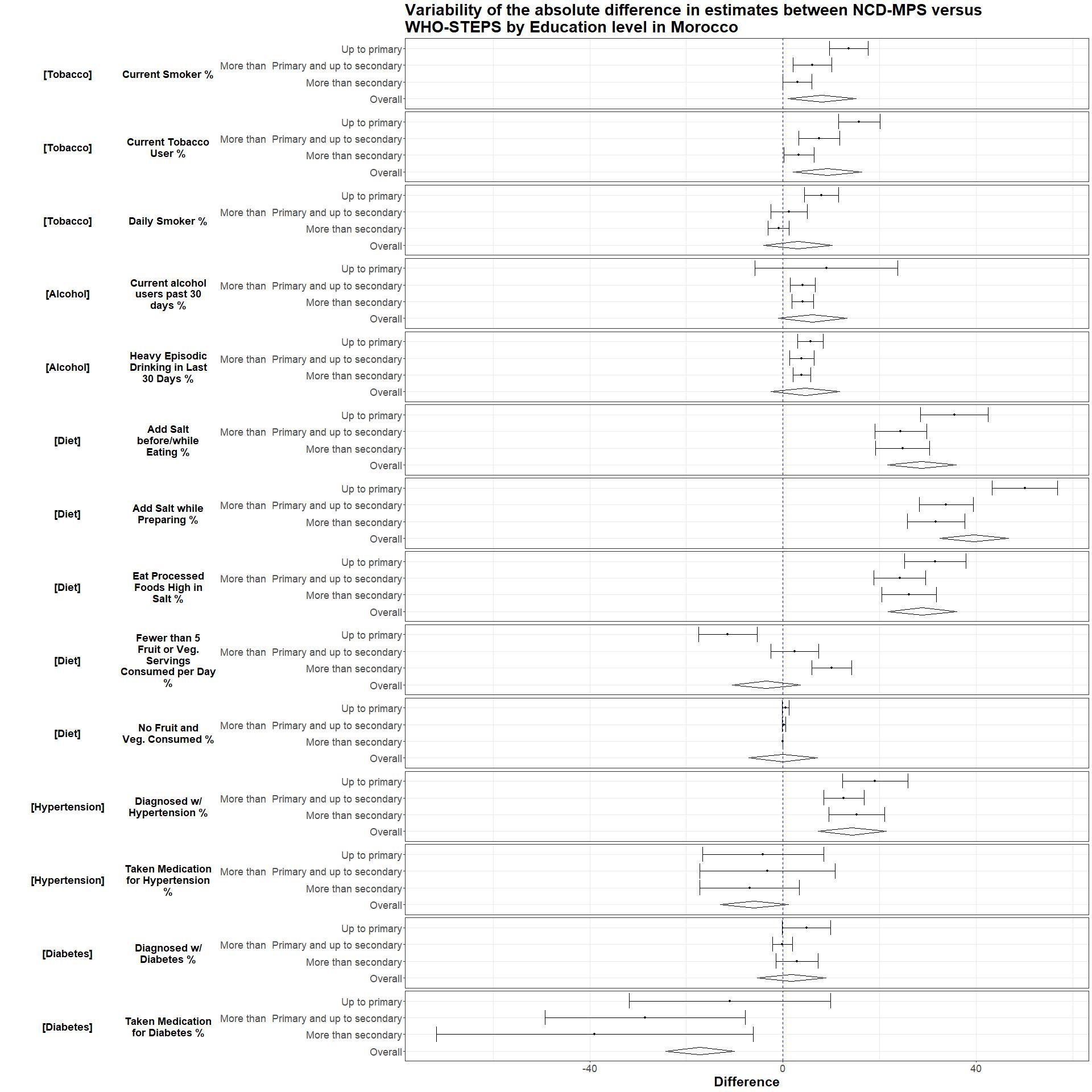


# **Supplementary Figure 16. Subgroup analysis of NCD indicators by education in Zambia**


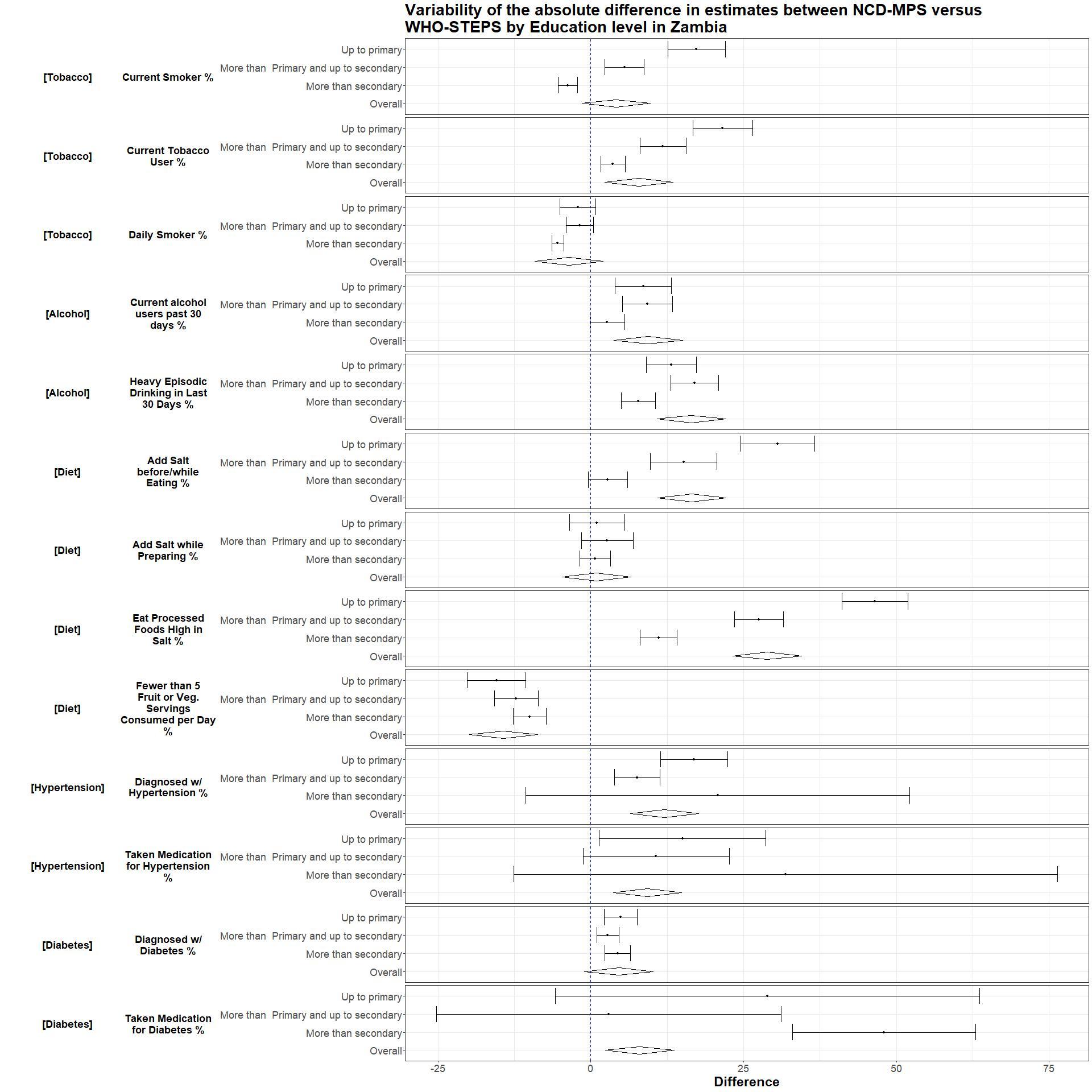


# **Supplementary Figure 17. Subgroup analysis of NCD indicators by education in Sri Lanka**


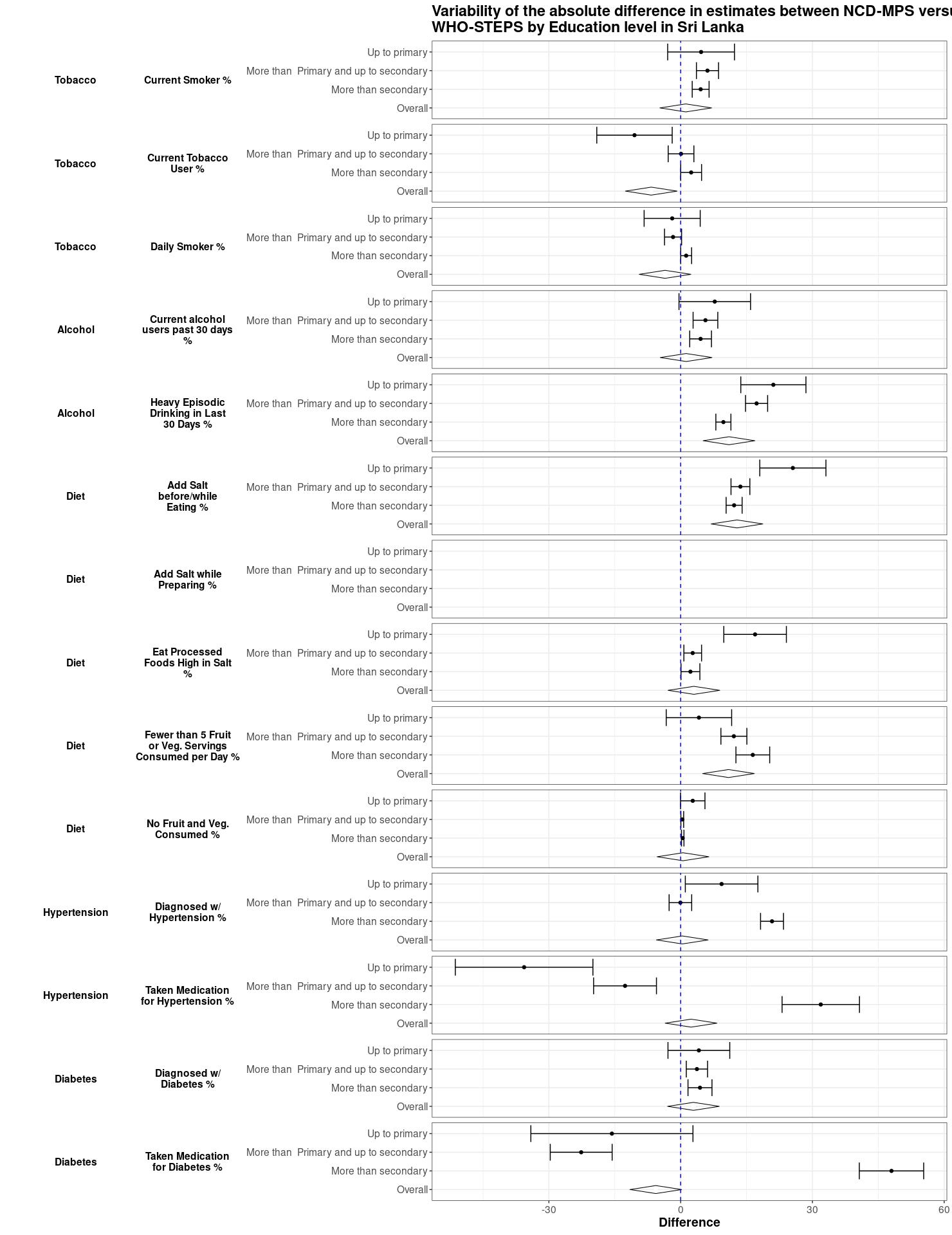


# **Supplementary Figure 18. Subgroup analysis of NCD indicators by education in Mumbai**


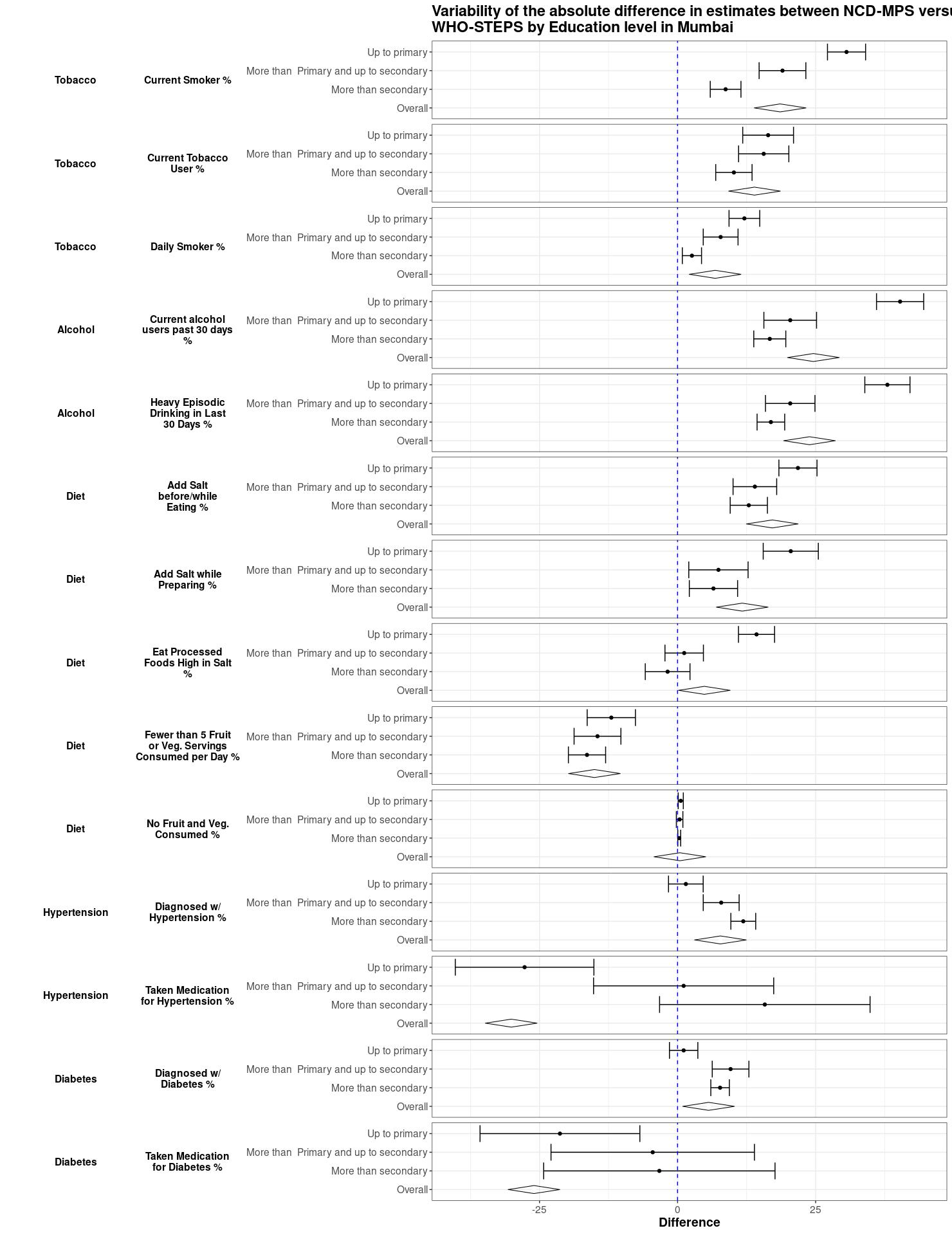

Supplement: online supplemental file 1 [file bmjgh-10-6-s001.docx]
